# Modulation of *Drosophila* post-feeding physiology and behavior by the neuropeptide leucokinin

**DOI:** 10.1371/journal.pgen.1007767

**Published:** 2018-11-20

**Authors:** Meet Zandawala, Maria E. Yurgel, Michael J. Texada, Sifang Liao, Kim F. Rewitz, Alex C. Keene, Dick R. Nässel

**Affiliations:** 1 Department of Zoology, Stockholm University, Stockholm, Sweden; 2 Department of Biological Sciences, Florida Atlantic University, Jupiter, FL, United States of America; 3 Department of Biology, University of Copenhagen, Universitetsparken 15, Copenhagen, Denmark; Katholieke Universiteit Leuven, BELGIUM

## Abstract

Behavior and physiology are orchestrated by neuropeptides acting as central neuromodulators and circulating hormones. An outstanding question is how these neuropeptides function to coordinate complex and competing behaviors. In *Drosophila*, the neuropeptide leucokinin (LK) modulates diverse functions, but mechanisms underlying these complex interactions remain poorly understood. As a first step towards understanding these mechanisms, we delineated LK circuitry that governs various aspects of post-feeding physiology and behavior. We found that impaired LK signaling in *Lk* and *Lk receptor* (*Lkr)* mutants affects diverse but coordinated processes, including regulation of stress, water homeostasis, feeding, locomotor activity, and metabolic rate. Next, we sought to define the populations of LK neurons that contribute to the different aspects of this physiology. We find that the calcium activity in abdominal ganglia LK neurons (ABLKs), but not in the two sets of brain neurons, increases specifically following water consumption, suggesting that ABLKs regulate water homeostasis and its associated physiology. To identify targets of LK peptide, we mapped the distribution of *Lkr* expression, mined a brain single-cell transcriptome dataset for genes coexpressed with *Lkr*, and identified synaptic partners of LK neurons. *Lkr* expression in the brain insulin-producing cells (IPCs), gut, renal tubules and chemosensory cells, correlates well with regulatory roles detected in the *Lk* and *Lkr* mutants. Furthermore, these mutants and flies with targeted knockdown of *Lkr* in IPCs displayed altered expression of insulin-like peptides (DILPs) and transcripts in IPCs and increased starvation resistance. Thus, some effects of LK signaling appear to occur via DILP action. Collectively, our data suggest that the three sets of LK neurons have different targets, but modulate the establishment of post-prandial homeostasis by regulating distinct physiological processes and behaviors such as diuresis, metabolism, organismal activity and insulin signaling. These findings provide a platform for investigating feeding-related neuroendocrine regulation of vital behavior and physiology.

## Author summary

Animals ranging from jellyfish to humans use multiple neuropeptides to orchestrate various aspects of behavior and physiology. A major question in biology is how animals are able to coordinate complex and competing behaviors to ensure maintenance of a stable internal environment. To address this, we delineated the functions of the neuronal pathways using the neuropeptide leucokinin (LK) in the fruit fly *Drosophila melanogaster*. We discovered that mutant flies lacking LK signaling exhibit defects in diverse but coordinated processes, including regulation of stress, water balance, gut function, activity, and metabolic rate. We also attribute these functions to different subsets of neurons that produce LK. Lastly, we show that this neuropeptide interacts with insulin signaling to affect stress tolerance and metabolism. This is of broad interest since stress, obesity and ensuing metabolic disorders, such as heart disease and diabetes, are immense problems in society. Our work provides a foundation for further investigation of neuroendocrine regulation of vital behavior and physiology associated with feeding.

## Introduction

Animals continuously adjust to changes in their external and internal environment [1–3] and a central question is how homeostatically regulated behaviors and physiological processes critical for survival interact. In metazoans, neuropeptides play important roles in orchestrating homeostasis by mediating neuromodulation in circuits of the CNS and acting on peripheral tissues as circulating hormones [4–6]. We ask here whether a neuroendocrine system, using a single neuropeptide, can play a role in modulating complex behavioral and physiological processes. The neuropeptide leucokinin (LK) in the fly *Drosophila* is an excellent candidate to study modulation at multiple levels because it is expressed in three small sets of neurons and has been implicated in several homeostatically regulated functions, including sleep, feeding, water balance and response to ionic stress [7–13].

Previous *in vitro* work has suggested that one important function of LK in adult *Drosophila* and several other insect species is to regulate fluid secretion in the Malpighian (renal) tubules (MTs), and, thus, to play an important role in water and ion homeostasis [9,14–17]. More recently, additional LK functions have been inferred from *in vivo* genetic experiments, such as roles in organismal water retention, survival responses to desiccation and starvation, subtle regulation of food intake, and chemosensory responses [10,13,18–21]. Furthermore, it has been shown that diminished LK signaling results in an increase in postprandial sleep [12] and impaired locomotor activity [11]. While we know that LK is critical for behavioral and physiological homeostasis, it is not clear how a relatively small population of less than 30 neurons can mediate diverse responses to environmental perturbation. Moreover, it remains unclear whether the different functions revealed are all part of a global orchestrating role of LK in which central and peripheral actions are coordinated at different levels. In the light of this, it is of interest to identify the functional roles of each of the three sets of LK neurons and to determine how these contribute to a coordinated modulation of homeostasis.

To determine the role of LK signaling in adult post-feeding physiology and behavior, we generated novel *Lk* and *Lkr* mutant flies. By testing these mutants in various feeding-related physiological and behavioral assays, we demonstrate that LK signaling regulates water homeostasis and associated stress, feeding, locomotor activity, and metabolic rate. Based on these data, we propose that the homeostatic roles of LK can be linked to the regulation of post-feeding physiology and behavior. The abdominal ganglion LK neurons (ABLKs), but not the two sets in the brain, display increased calcium-signaling activity in response to rehydration (drinking) following desiccation. Next, to reveal novel targets of LK peptide, we mapped the distribution of *Lkr* expression. Using two independent *Lkr-GAL4* lines to drive expression of GFP, we show that *Lkr* is expressed in various peripheral tissues, including the gut, Malpighian tubules and chemosensory cells, which comports well with the functions suggested by the mutant analysis. In addition, the expression of the *Lkr* in the insulin-producing cells (IPCs) and the phenotypes seen after targeted receptor knockdown in these cells indicate interaction between LK and insulin signaling. Thus, the three different populations of LK neurons use LK to modulate post-prandial physiology by acting on different targets in the CNS, as well as cells of the renal tubules and intestine.

## Results

### Generation and analysis of *Lk* and *Lkr* mutant flies

**Fig 1:**
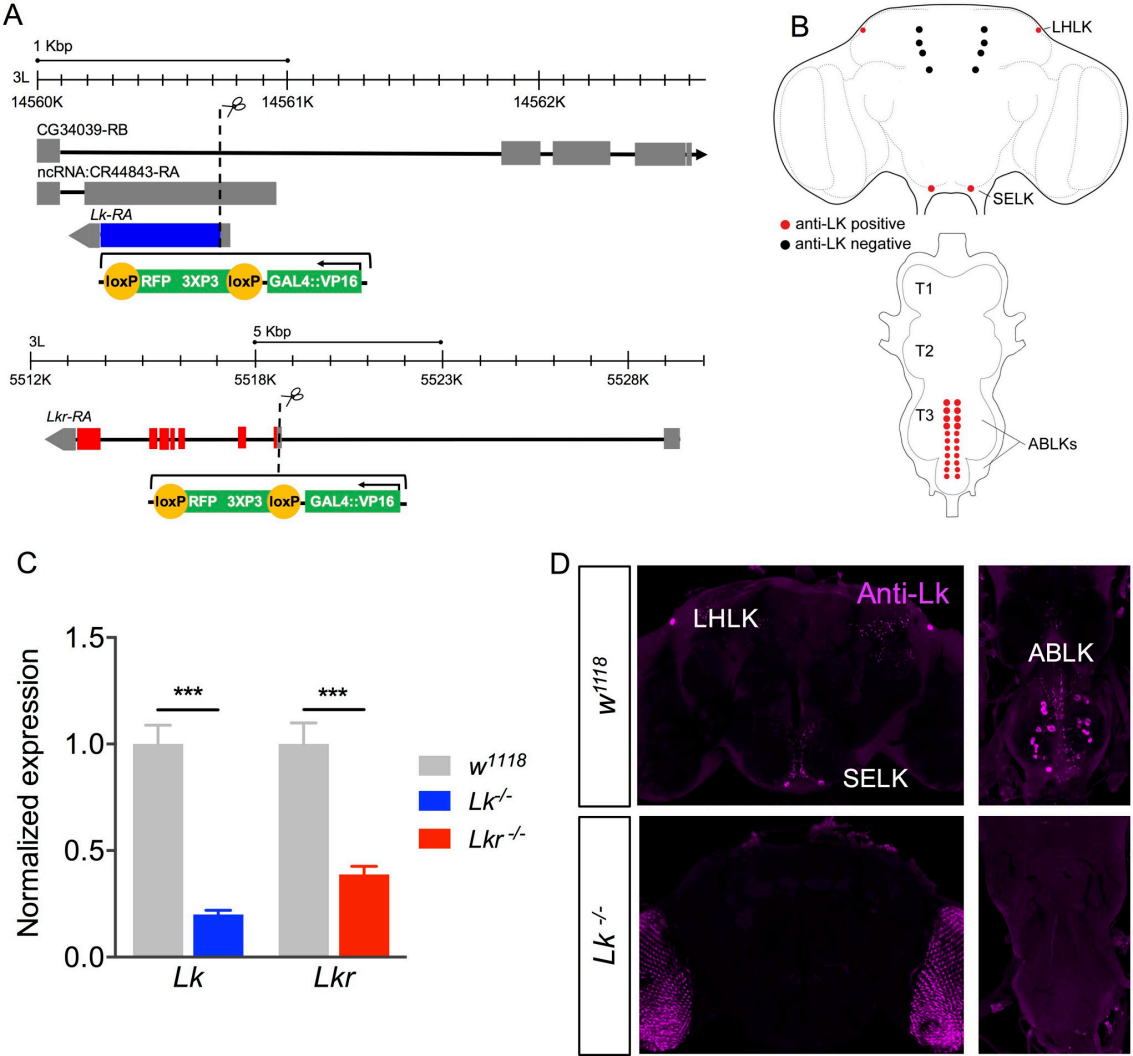
Generation of *Lk* and *Lkr GAL4* knock-in mutants. **(A)** Schematics of the *Lk* and *Lkr* gene loci and the locations of construct insertion to generate *GAL4* knock-in mutants. Note that *CG34039* and ncRNA represent predictions for the presence of coding and non-coding genes in the same chromosome and overlapping location as *Lk*. However, there is no evidence that they are functional. Potentially, these two genes are encoded on the sense strand while *Lk* is on the anti-sense strand. **(B)** A schematic of the adult CNS showing the location of LK-expressing neurons [based on [7,8,10]]. LHLK, lateral horn LK neuron; SELK, subesophageal ganglion LK neuron; ABLK, abdominal LK neuron, T1 –T3, thoracic neuromeres. **(C)** Quantitative PCR shows a significant reduction in *Lk* and *Lkr* transcripts in *Lk* and *Lkr* homozygous mutants, respectively. (*** p < 0.001 as assessed by unpaired *t* test). **(D)** LK-immunoreactivity is completely abolished in the brain and ventral nerve cord of *Lk* mutants.

**Fig 2:**
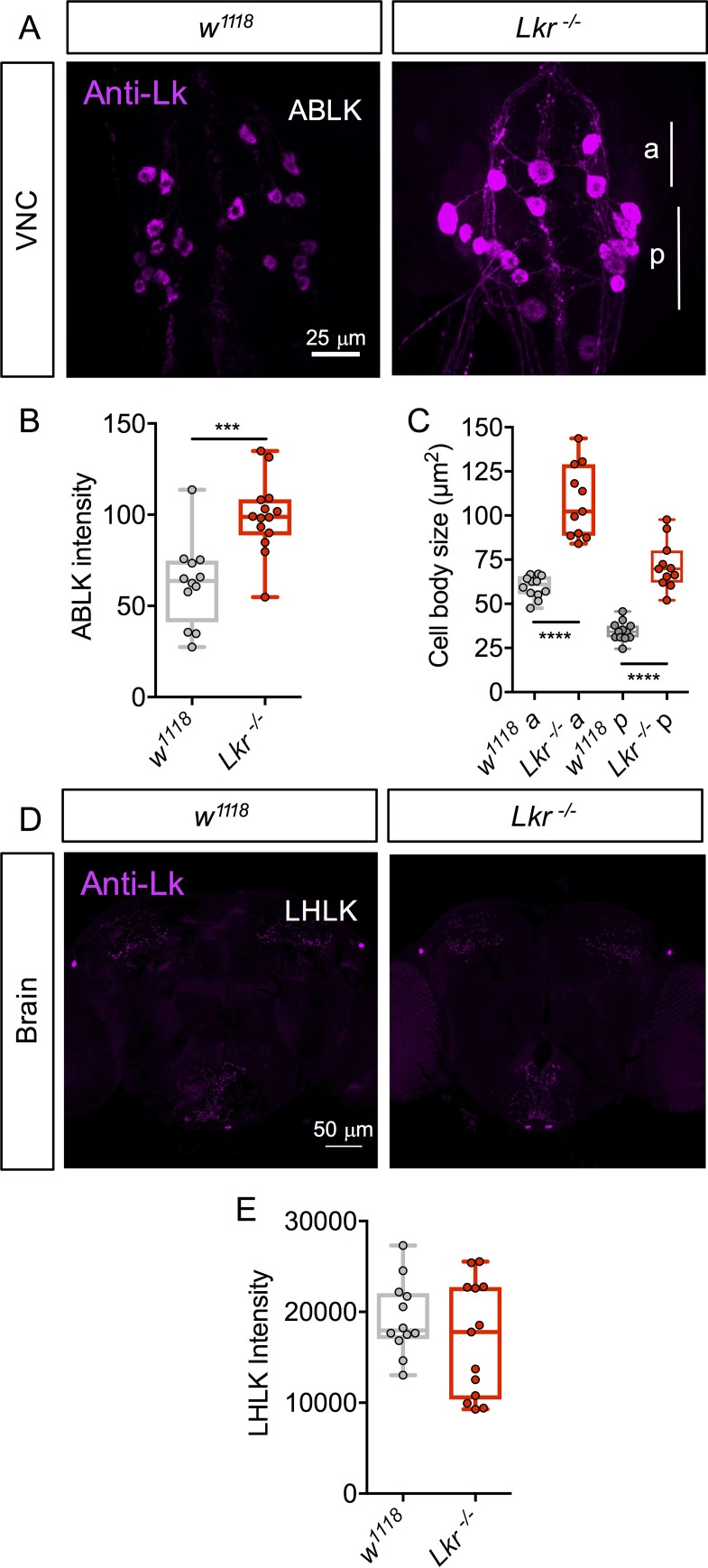
LK cell body size and peptide levels in *Lkr* mutants. **(A)** LK-immunoreactivity in abdominal LK neurons (ABLKs) of *Lkr* mutant and control flies. **(B)** Staining intensity and **(C)** cell-body size of both the anterior (a) and posterior (p) ABLKs is increased in *Lkr* mutants compared to control flies. We separated the two cell groups here since the anterior (and larger) ABLKs are derived post-embryonically (during metamorphosis), and the posterior ones are functional already in the larva (see [22]). **(D)** LK-immunoreactivity in brain lateral horn LK neurons (LHLKs) of *Lkr* mutant and control flies. **(E)** The intensity of LK staining is unaltered in *Lkr* mutants. (**** p < 0.0001 as assessed by one-way ANOVA followed by Tukey’s multiple comparisons test for **C** and *** p < 0.001 as assessed by unpaired *t* test for **B**).

To investigate the role of Lk signaling in modulation of feeding-associated physiology and behavior, we utilized CRISPR-Cas9 gene editing to generate GAL4 knock-in mutants for *Lk* and *Lkr* (Fig 1A). First, we tested the efficiency of the *Lk* and *Lkr* mutants by quantitative real-time PCR (qPCR) and immunolabeling. In qPCR experiments, we found an 80% reduction of *Lk* expression, whereas *Lkr* mRNA was reduced by about 60% (Fig 1C), confirming the efficacy of these gene-edited mutants for *Lk and Lkr* (residual expression presumably reflects some level of transcriptional read-through of the inserted *GAL4* cassette). In the homozygous *Lk* mutants, LK immunolabeling is completely abolished in all cells of the CNS (Fig 1B and 1D), establishing that *Lk* mutants do not produce a functional peptide. To verify that signaling by LKR is disrupted in *Lkr* mutants, we measured LK peptide levels by immunolabeling. The rationale for this was that we predicted that *Lkr* mutant flies would compensate for the diminished receptor expression, for instance in MTs, by increasing production of the peptide in neurosecretory cells to maintain homeostasis. Indeed, LK immunolabeling was elevated in the abdominal LK neurons (ABLKs) (Fig 2A and 2B), and the cell bodies of these neurons were also enlarged (Fig 2C), probably due to the increased peptide production [see [22]]. Interestingly, LK immunolabeling in the lateral horn LK (LHLK) neurons of the brain does not change in *Lkr* mutant flies (Fig 2D and 2E), suggesting these neurons are not subjected to autoregulatory feedback. Thus, LK levels are differentially regulated in neurons of the brain versus those of the abdominal ganglion, and there appears to be feedback between receptor and peptide expression in abdominal ABLK neurons of *Lkr* mutant flies. A possible explanation for this is that the ABLKs are neurosecretory cells that target peripheral tissues such as MTs with hormonal LK (see [10]) and periphery-to-CNS feedback may be critical for homeostatic regulation.

**Fig 3:**
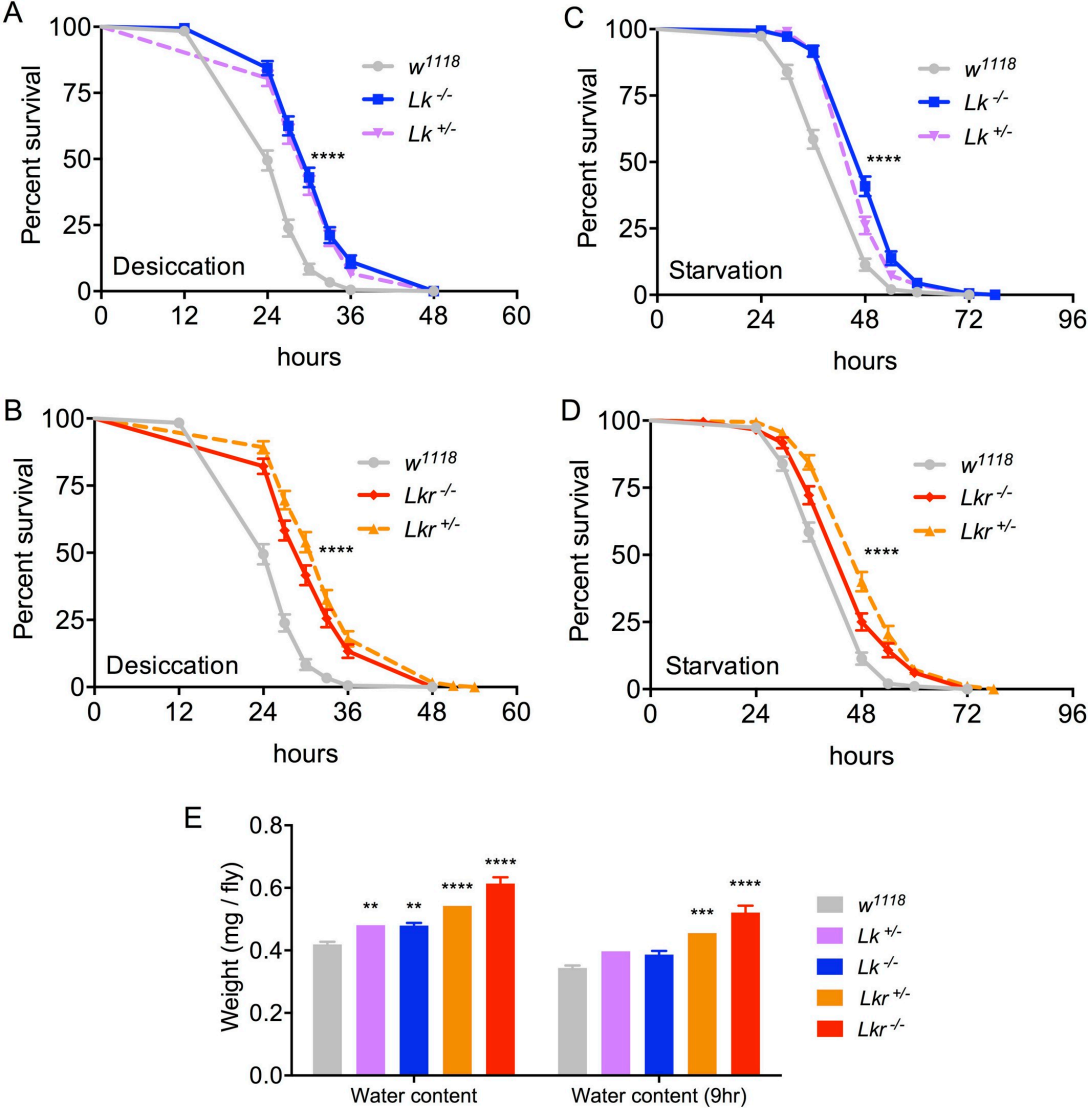
*Lk* and *Lkr* mutants have altered stress resistance and water content. Survival under desiccation is increased in both **(A)** *Lk* and **(B)** *Lkr* mutants. Survival under starvation is also increased in both **(C)** *Lk* and **(D)** *Lkr* mutants. Data are presented in survival curves, and the error bars represent standard error (**** p < 0.0001, as assessed by Log-rank (Mantel-Cox) test). **(E)** Hydrated and 9-hour-desiccated (9 h) *Lk* and *Lkr* mutant flies show increased water content compared to control flies. (** p < 0.01, *** p < 0.001, **** p < 0.0001 as assessed by one-way ANOVA followed by Tukey’s multiple comparisons test).

Having validated the loss of function in the *Lk* and *Lkr* mutants, we tested them for phenotypes that have been previously associated with LK signaling. Previous studies, *in vitro* or using different types of manipulations, have demonstrated a role of LK signaling in MT secretion [14,17] and a possible secondary effect of this on desiccation and starvation resistance [10,19,21]. We therefore recorded survival of *Lk* and *Lkr* mutant flies maintained under desiccation and starvation conditions. Both homozygous and heterozygous *Lk* (*Lk*-GAL4^CC9^) and *Lkr* (*Lkr*-GAL4^CC9^) mutants, survived longer under these stresses (Fig 3A–3D). To determine whether changes in water content contributed to these survival differences, we assayed flies for their water content under normal conditions and after 9 hours of desiccation. As expected, *Lk* and *Lkr* mutant flies contained more water than control flies did under normal conditions as well as after desiccation (Fig 3E). Therefore, loss of Lk/Lkr signaling promotes water retention and improves survival under desiccation conditions.

**Fig 4:**
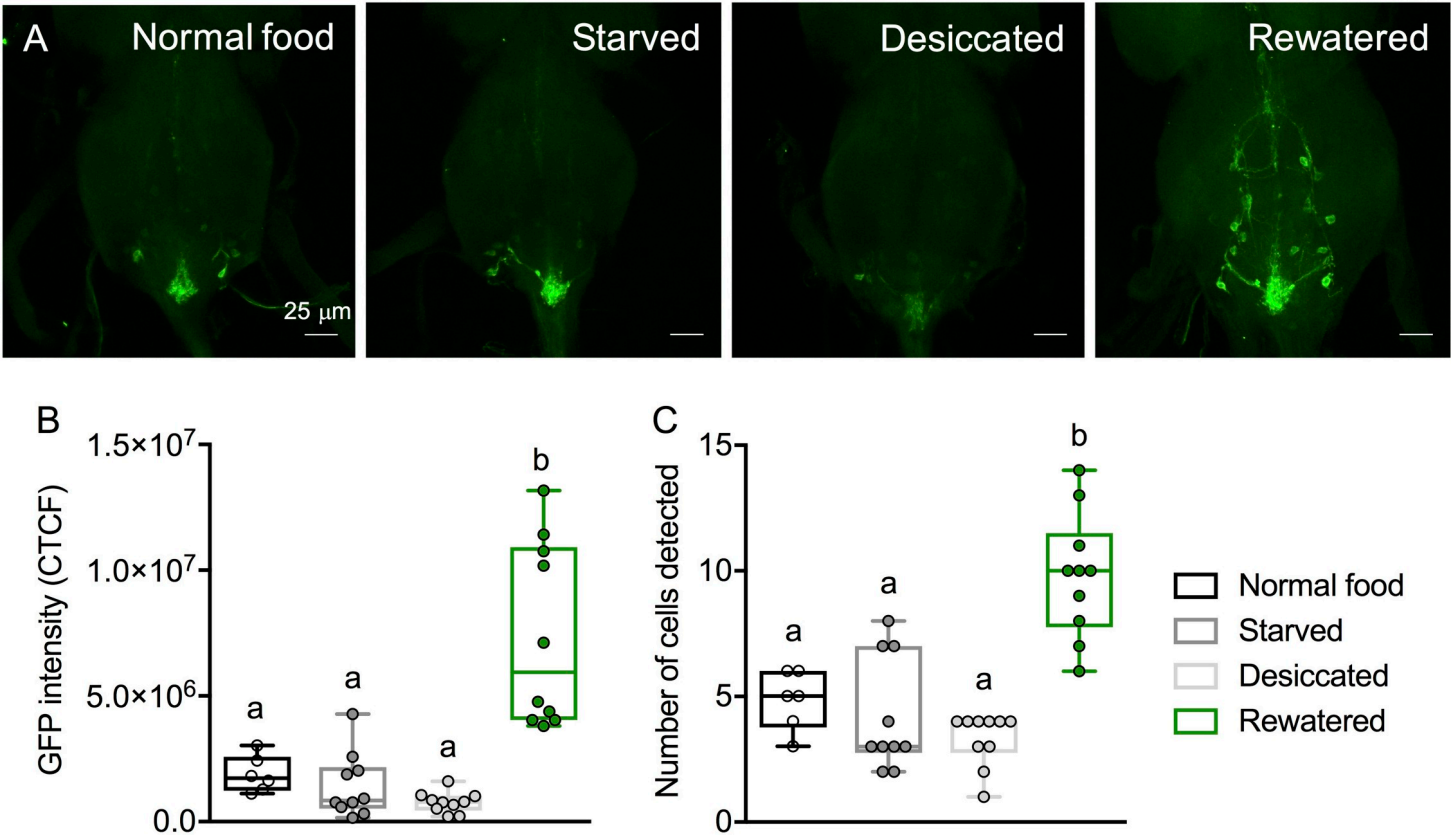
Calcium activity of ABLKs under nutritional and osmotic stress. **(A)** The calcium activity of ABLKs, as measured using CaLexA [23], is low in flies that have been starved, desiccated, or incubated on normal artificial food but increased in flies that have been rewatered (desiccated and then incubated on 1% agar). **(B)** The GFP intensity of ABLKs is increased in rewatered flies compared to other conditions. **(C)** The number of ABLKs that could be detected is higher in rewatered flies compared to other conditions. (assessed by one-way ANOVA followed by Tukey’s multiple comparisons test).

Next, we asked which of the LK neurons might be responsible for these effects on water homeostasis and associated stresses. To determine which of the LK neurons display activity-dependent changes in response to starvation, desiccation, and/or water ingestion we monitored the calcium activity of LK neurons using the CaLexA system [23]. By expressing the CaLexA sensor with the *Lk-GAL4* driver, we found that only the ABLKs, but not the LK neurons in the brain, were activated following re-watering (drinking) (Fig 4A). The activation of ABLKs can be seen as increased GFP intensity as well as a greater number of detectable cells (Fig 4B and 4C). Moreover, these cells did not display activation when the flies are placed under starvation, desiccation, or on a standard diet. These results further support the role of ABLKs in the regulation of water homeostasis.

**Fig 5:**
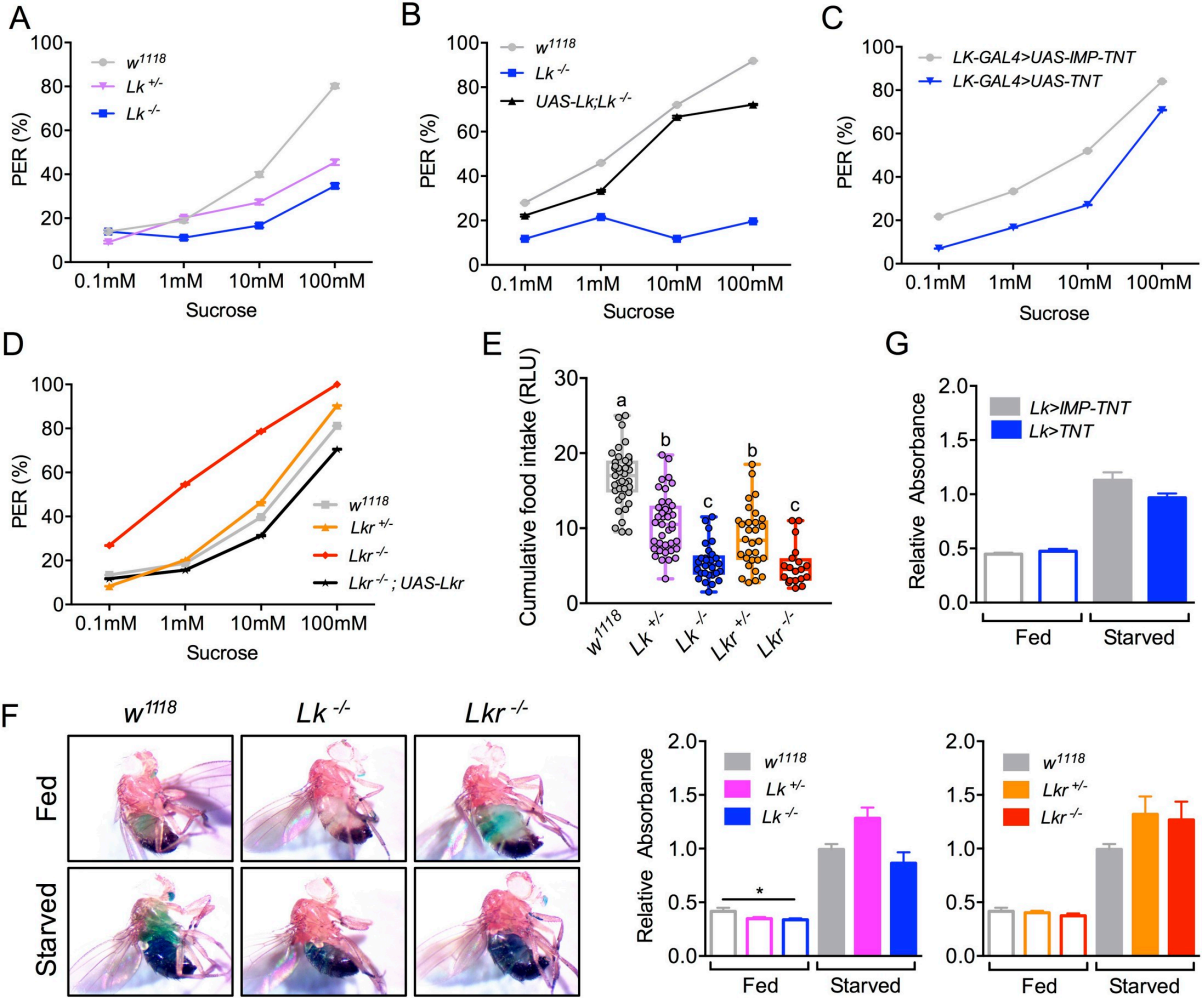
*Lk* and *Lkr* mutants show varying phenotypes in different feeding assays. **(A)** Both the homozygous and heterozygous *Lk* mutants show decreased motivation to feed in proboscis extension reflex (PER) and this phenotype could be rescued in **(B)** the homozygous flies. **(C)** Targeted expression of tetanus toxin (to block synaptic transmission) in *Lk* neurons using *Lk-GAL4* also caused a decrease in PER. **(D)** Interestingly, *Lkr* mutants show increased motivation to feed, which could be rescued to control levels by driving *UAS-Lkr* with *Lkr-GAL4*^*CC9*^. See S1 Table for the statistics of graphs A-D. **(E)** Both the *Lk* and *Lkr* mutants show decreased long-term food intake as measured using the capillary feeding (CAFE) assay. Moreover, the homozygous mutants feed significantly lower than the heterozygous mutants (assessed by one-way ANOVA followed by Tukey’s multiple comparisons test). **(F)** Starved and fed *Lk* and *Lkr* mutants do not show any differences in short-term feeding compared to control flies as measured using a blue-dye feeding assay (assessed by one-way ANOVA). **(G)** Expression of tetanus toxin in *Lk* neurons with *Lk-GAL4* also has no effect on short-term feeding.

Having established a role for LK signaling in water homeostasis and activation of ABLKs in response to water intake, we asked whether LK signaling might affect other aspects of feeding-associated physiology and behavior. Hence, we examined *Lk* and *Lkr* mutants in various assays to monitor feeding propensity and food intake over different time scales. First, we tested the *Lk* and *Lkr* mutants for the strength of the proboscis extension reflex (PER) in response to different sucrose concentrations (Fig 5A–5D and S1 Table) to quantify gustation and/or the motivation to feed. The *Lk* mutant flies displayed a reduced PER (Fig 5A) and this phenotype was rescued by re-expressing the peptide by UAS-*Lk* in the homozygous *GAL4*-insertion mutants (Fig 5B). This reduction in PER was also seen after inhibition of LK neurons by targeted expression of UAS-*Tetanus toxin* (*TNT)* (Fig 5C). However, *Lkr* mutant flies displayed the opposite behavior, showing increased PER that could also be rescued by UAS-*Lkr* expression (Fig 5D). This suggests a role for LK signaling in gustation (see also [8,18]), but the opposite behavior seen in peptide and receptor mutant flies is difficult to explain. Maybe in the gustatory system LK acts through an alternative receptor type or different coupling to downstream signaling pathway. Next, we assayed for long-term defects in feeding by examining the mutants in a modified capillary feeding (CAFE) assay (Fig 5E). Both, *Lk* and *Lkr* mutants exhibited a decrease in food intake compared to controls, with the homozygous mutants displaying a much larger decrease than the heterozygous ones (Fig 5E). Finally, we used an assay for short-term feeding (over 30 min), in which the amount of ingested blue-dyed food was measured in fly homogenates to determine differences in meal sizes. In this assay, there was no difference in food intake between mutant flies and controls, either in starved or fed conditions (Fig 5F). This lack of effect was also seen when the LK neurons were inhibited by targeted expression of UAS-*TNT* (Fig 5G). Therefore, LK neurons seem to regulate the propensity of animals to initiate reflexive feeding, without affecting total meal volume in the short-term, but probably contributes to reduced food intake over longer time frames.

**Fig 6:**
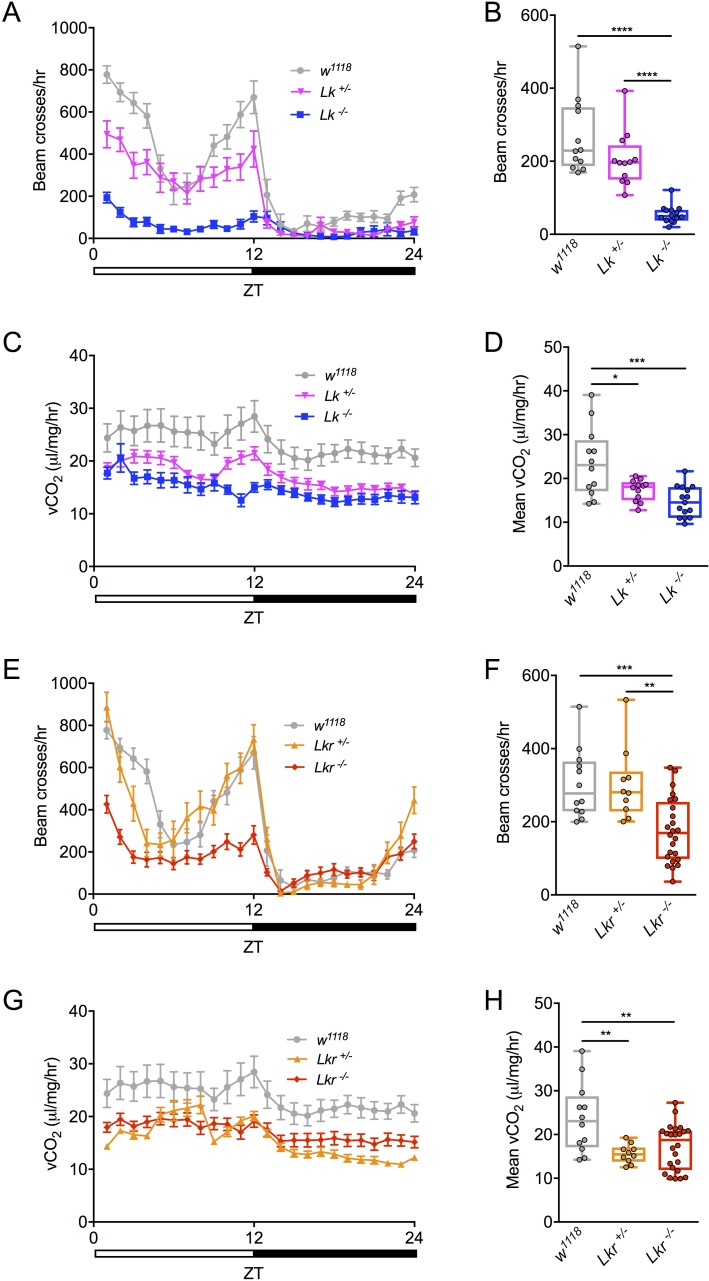
Total activity and metabolic rate is lowered in individual *Lk* and *Lkr* mutants. **(A)** Locomotor activity pattern of individual *Lk* homozygous and heterozygous mutants measured over 24 hours. **(B)** Total locomotor activity of *Lk* mutants is lowered compared to control flies. **(C)** Metabolic rate rhythms of individual *Lk* homozygous and heterozygous mutants measured over 24 hours. **(D)** Average metabolic rate of *Lk* mutants is lowered compared to control flies. **(E)** Locomotor activity pattern of individual *Lkr* homozygous and heterozygous mutants measured over 24 hours. **(F)** Total locomotor activity of *Lkr* mutants is lowered compared to control flies. **(G)** Metabolic rate rhythms of individual *Lkr* homozygous and heterozygous mutants measured over 24 hours. **(H)** Average metabolic rate of *Lkr* mutants is lowered compared to control flies. (* p < 0.05, ** p < 0.01, *** p < 0.001, **** p < 0.0001 as assessed by one-way ANOVA).

Physical activity and metabolic rate are acutely regulated by food availability and environmental stress. To determine whether LK regulates these processes we simultaneously recorded animal activity and metabolic rate using stop-flow indirect calorimetry [24]. Single *Lk* and *Lkr* mutant flies were tested for locomotor activity and metabolic rate (vCO_2_) over a 24-hour period. The *Lk* mutants displayed reduced locomotor activity, with homozygotes displaying almost no morning or evening activity peaks (Fig 6A and 6B). The metabolic rate of these mutant flies was also reduced over the entire period of observation (Fig 6C and 6D). The *Lkr* mutants displayed a similar reduction in both locomotor activity and metabolic rate, except that the heterozygotes displayed no change in locomotor activity (Fig 6E–6H). We also used the standard *Drosophila* activity monitor system (DAMS) to verify our locomotor-activity results from the above setup. Indeed, we obtained results similar to those above, with *Lk* and *Lkr* mutants displaying reduced activity (S1A and S1B Fig). Taken together, these findings suggest that disruption of Lk-signaling leads to dysregulation of metabolic rate and altered locomotor activity.

### Identification of central and peripheral targets of LK

The expression of *Lk* and *Lkr* in the central nervous system (CNS) and periphery raises the possibility that distinct neuronal populations or neural circuits regulate different behaviors. The *Lk* and *Lkr-GAL4* knock-in mutants (*GAL4*^*CC9*^) that we generated using CRISPR-Cas9 gene editing enable simultaneous knockdown and visualization of the distribution of peptide- and receptor-gene expression in different tissues. Since the *GAL4* is inserted within the gene itself, the retention of all the endogenous regulatory elements should in theory allow *GAL4* expression to mimic that of the native *Lk* and *Lkr*. Indeed, the *Lk*-*GAL4*^*CC9*^ expression observed (S2 Fig) is very similar to that seen in earlier reports using conventional *Lk*-*GAL4* lines [8,13]. With a few exceptions, the pattern of *Lk*-*GAL4*^*CC9*^ expression also matches that of LK immunolabeling (S2C and S2D Fig). Notably, a set of 5 pairs of GFP-labeled lateral neurosecretory cells does not display LK immunolabeling in third instar larvae or adult flies (S2C and S3A Figs). These neurons are known as ipc-1 and ipc-2a, and they co-express ion transport peptide (ITP), short neuropeptide F (sNPF) and *Drosophila* tachykinin (DTK) [25,26].

**Fig 7:**
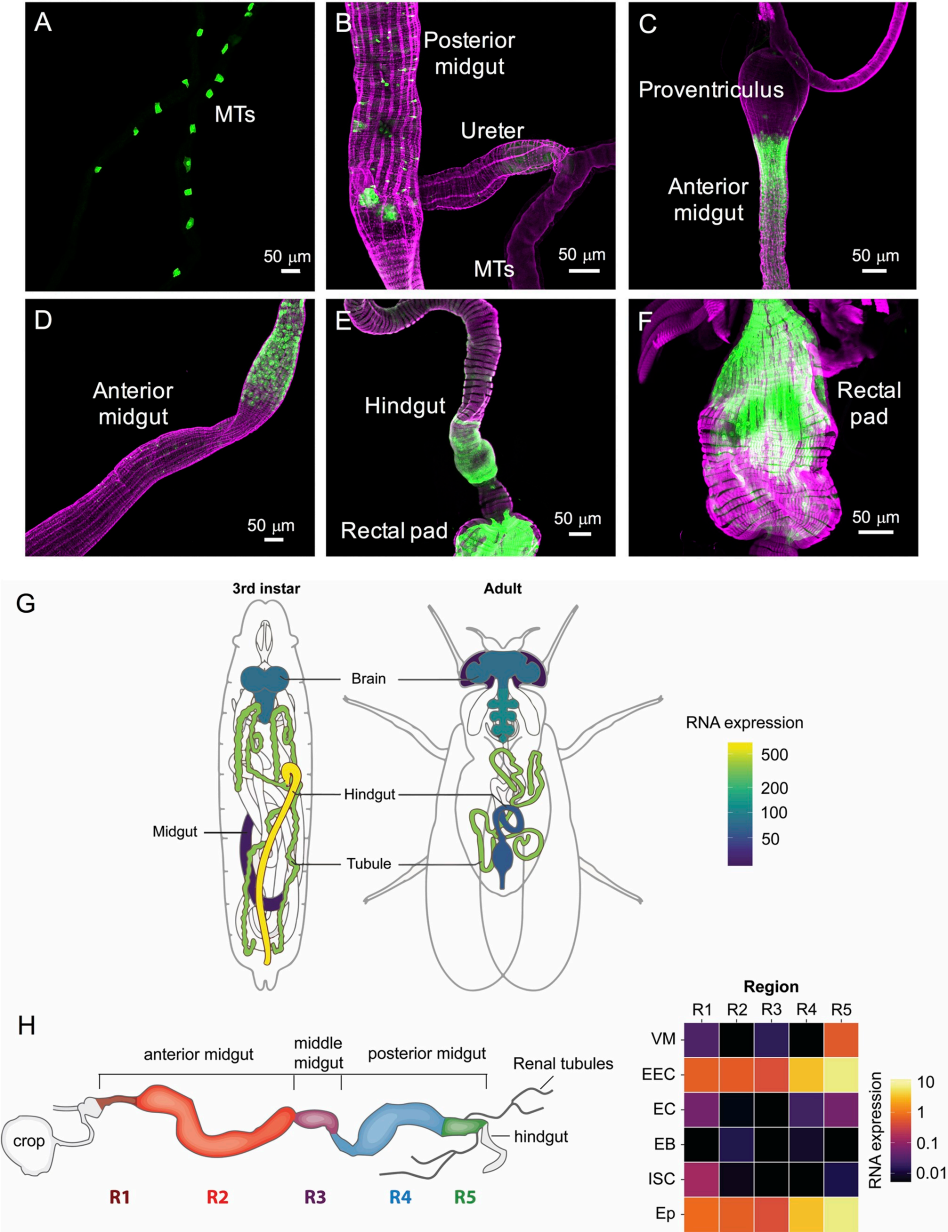
*Lkr* is expressed in the adult gut and Malpighian tubules. *Lkr-GAL4*^*CC9*^ drives GFP (*pJFRC81-10xUAS-Syn21-myr*::*GFP-p10*) expression in the adult **(A)** stellate cells in Malpighian tubules, **(B)** enteroendocrine cells in the posterior midgut, **(C and D)** anterior midgut, **(E)** hindgut, and **(F)** rectal pad. Muscles (F-actin filaments) in all the preparations (except B) have been stained with rhodamine-phalloidin (magenta). Note the expression of GFP in hindgut and rectal pad muscles. **(G)** Schematics of third instar larvae and adult fly showing the expression of *Lkr*. (Data from FlyAtlas.org, [28]). **(H)** A schematic of the adult gut and heat map showing expression of *Lkr* in different regions of the gut (R1 to R5) and its various cell types (VM, visceral muscle; EEC, enteroendocrine cell; EC, enterocyte; EB, enteroblast; ISC, intestinal stem cell; Ep, epithelium. Data was mined using Flygut-*seq* [29].

Since the cellular expression pattern of *Lkr* in *Drosophila* is poorly understood we utilized our *Lkr*-*GAL4*^*CC9*^ line to drive GFP-expression and analyzed CNS and peripheral tissues. We compared the expression of our *Lkr*-*GAL4*^*CC9*^ to that of another *Lkr*-*GAL4* (*Lkr-GAL4*::*p65*) generated using a BAC clone as described previously [27] and found overlapping expression patterns between the two drivers. In the periphery, the stellate cells of the MTs express *Lkr*-*GAL4*^*CC9*^ (Fig 7A) as expected from earlier work that demonstrated functional expression of the Lkr in these cells [14,17]. Furthermore, *Lkr*-*GAL4*^*CC9*^ driven GFP was detected in endocrine cells of the posterior midgut (Fig 7B), in the anterior midgut (Fig 7C and 7D), and in muscle fibers of the anterior hindgut and rectal pad (Fig 7E and 7F). *Lkr-GAL4*^*CC9*^*>GFP* expression was also present in peripheral neurons (S4A Fig), the dorsal vessel, as well as axons innervating it (S4A Fig), and sensory cells of the legs, mouthparts, and anterior wing margin (S4B–S4D Fig). In third instar larvae, we could also detect *Lkr*-*GAL4*^*CC9*^ expression in the stellate cells of the MTs (S5A and S5D Fig), in the ureter (S5A Fig), in muscle fibers of the gastric caeca, midgut and hindgut (S5A–S5C Fig), as well as in the endocrine cells of the midgut (S5B and S5C Fig). The BAC-engineered *Lkr-GAL4* had a much sparser expression pattern, with GFP detected in stellate cells of larval (S6A Fig) and adult (S6C–S6E Fig) MTs, and in the larval hindgut (S6B Fig). Interestingly, the shape of the stellate cells in adults varied between cuboidal and the more typical star-shaped morphology (S6C and S6D Fig).

In general, the expression of the BAC/promoter fusion line is sparser than the new *Lkr-GAL4*^*CC9*^ line, but both are in agreement with available immunolabeling data on the MTs (S5D and S6E Figs), suggesting that they largely recapitulate the endogenous receptor expression pattern. To further validate the authenticity of the GFP expression in the periphery, we examined *Lkr* expression in two publicly available resources for gene expression, FlyAtlas [28] and Flygut-*seq* [29]. FlyAtlas reveals that *Lkr* is expressed in the larval and adult hindgut, MTs and CNS (Fig 7G). Moreover, the Flygut-*seq* data base shows that *Lkr* is expressed in enteroendocrine cells of the midgut, in visceral muscles near the hindgut, and in the gut epithelium [29] (Fig 7H). Thus, the transcript expression data correlate well with the GAL4 expression pattern.

**Fig 8:**
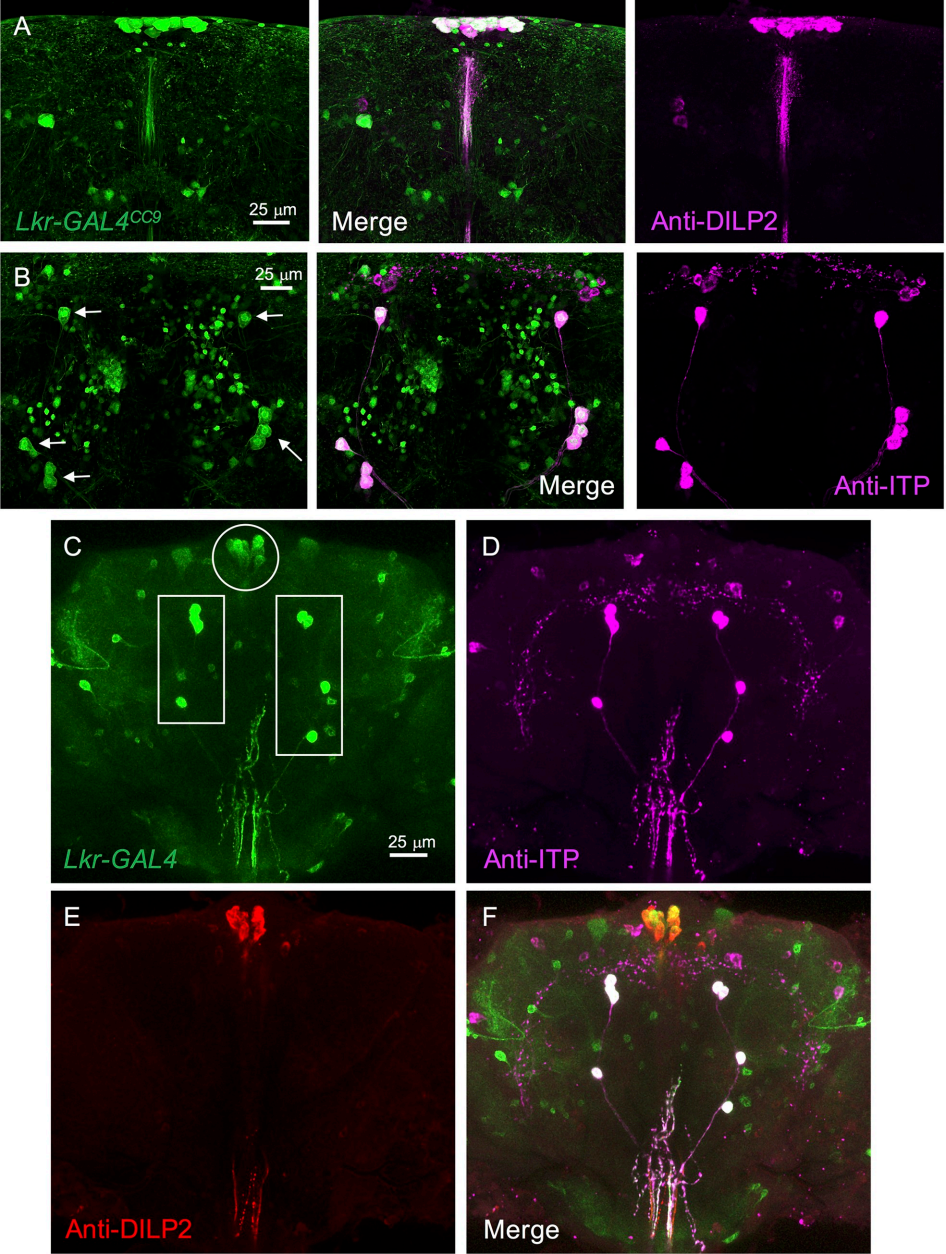
*Lkr* is expressed in identified peptidergic neurosecretory cells of the adult brain. *Lkr-GAL4*^*CC9*^ drives GFP (*pJFRC81-10xUAS-Syn21-myr*::*GFP-p10*) expression in **(A)** insulin-producing cells (labeled with anti-DILP2 antiserum) and **(B)** ion transport peptide (ITP)-producing lateral neurosecretory cells in the brain (labeled with anti-ITP antiserum; indicated by arrows). **(C)** *Lkr-GAL4* drives GFP (*UAS-mCD8;;GFP*) expression in the adult **(D and F)** ITP-producing cells (indicated by the white boxes in panel C) and **(E and F**) insulin-producing cells (indicated by the white circle in panel C).

**Fig 9:**
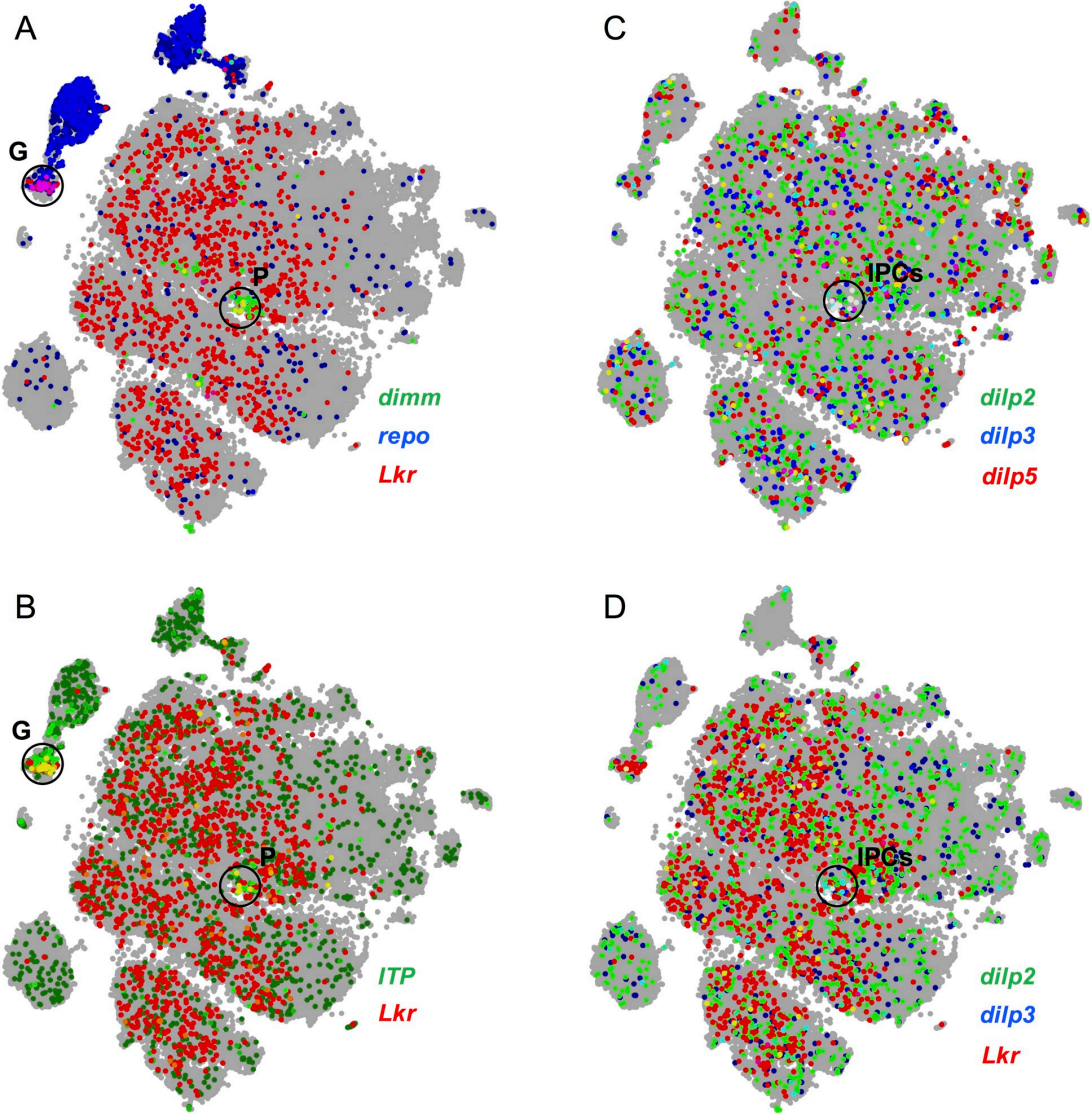
*Lkr* is coexpressed with peptidergic and glial markers. Mining the single-cell transcriptome atlas of the *Drosophila* brain reveals that *Lkr* is coexpressed with **(A)** *repo* (glial marker; cell cluster marked G) and *dimm* (peptidergic cell marker; cell cluster marked P). **(B)** Within both the glial and peptidergic cell clusters, *Lkr* is coexpressed with ITP. Within the peptidergic cell cluster, **(C)** insulin-producing cells expressing *DILP2*, *3* and *5* could be identified (cluster marked IPCs), a subset of which express *Lkr* **(D)**. Data was mined using Scope (http://scope.aertslab.org) [30]. In both **(C)** and **(D)**, cells expressing all three genes are colored in white.

The expression pattern of *Lkr*-*GAL4*^*CC9*^ and the *Lkr*-*GAL4* also matched well within the brain. Both GAL4 lines drive GFP expression in a relatively large number of neurons in the larval (S3B and S7A Figs) and adult CNS (S7B–S7C and S8 Figs), but we focus here on two sets of identified peptidergic neurons in the brain (Fig 8). Both *Lkr*-*GAL4*^*CC9*^ and *Lkr-GAL4*, drove GFP expression in the brain IPCs, as identified by anti-DILP2 staining, and in the 5 pairs of brain ipc-1/ipc-2a cells, that display anti-ITP staining (Fig 8). This receptor expression is supported by analysis of a single-cell transcriptome dataset of the entire *Drosophila* brain [30], which reveals coexpression between *Lkr* and *DILP2*, *3* and *5*, as well as *Lkr* and *ITP* (Fig 9). The data set shows that *Lkr* is widely expressed in the *Drosophila* brain, with transcripts expressed in cells of various clusters, including the peptidergic cell cluster (marked with *dimm*) and the glial cell cluster (marked with *repo*) (Fig 9A). Within the peptidergic cell cluster, *Lkr* is coexpressed with *ITP* (Fig 9B) and in IPCs along with *DILP2*, *3* and 5 (Fig 9C and 9D). Our receptor expression data further emphasizes the important interplay between LK signaling within the CNS and systemic LK action that targets several peripheral tissues, which together modulate feeding-associated physiology and behavior.

**Fig 10:**
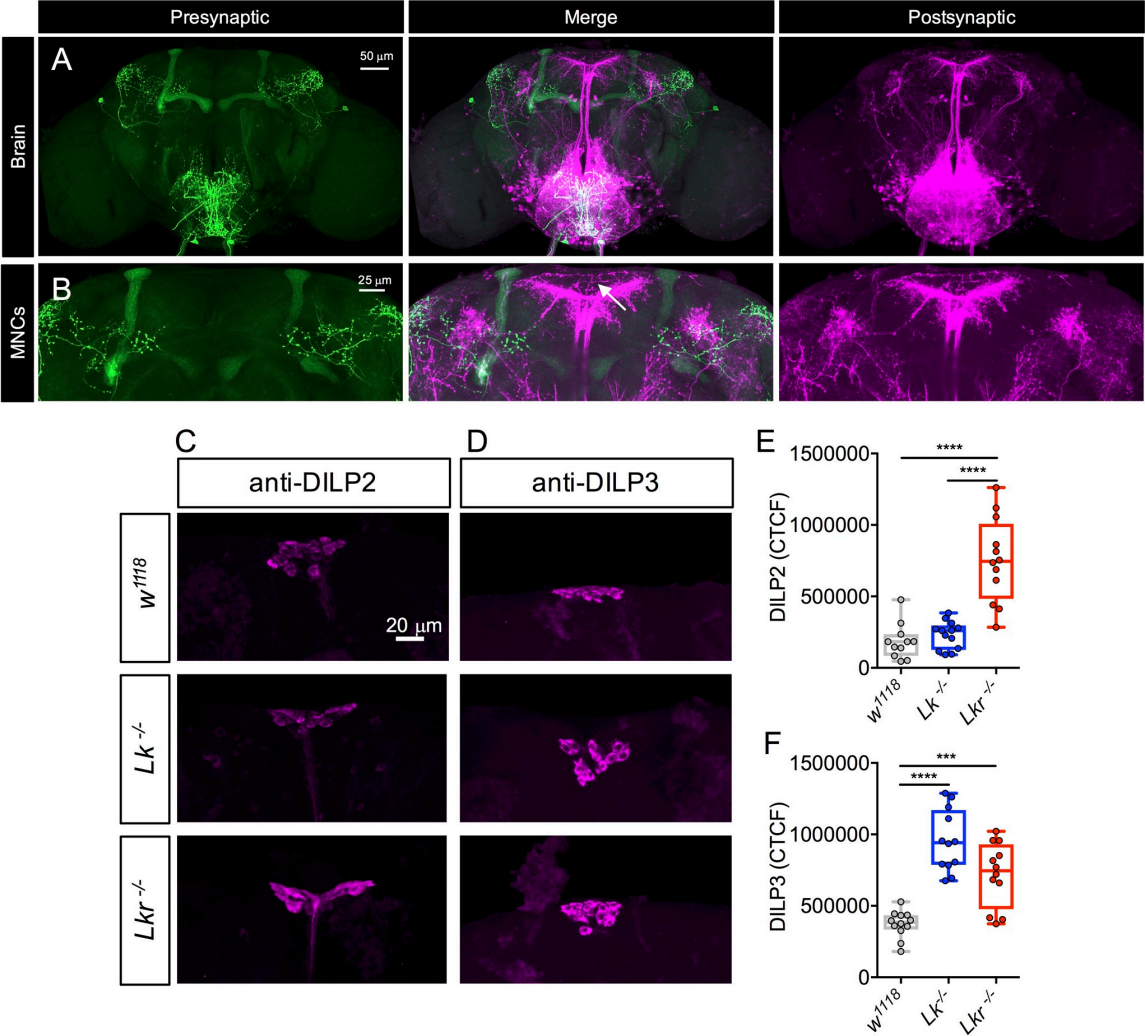
Anatomical and functional interactions between LK and insulin signaling. **(A)** Expression of *trans*-Tango components [31] using *Lk-GAL4* (from K. Asahina and D. Anderson) generates a presynaptic signal (labeled with anti-GFP antibody) in the subesophageal ganglion (SEG) and the lateral horn, and a postsynaptic signal (labeled with anti-HA antibody) in the SEG and pars intercerebralis. **(B)** Higher magnification of the SEG showing the presynaptic signals and the lack of post-synaptic signal in median neurosecretory cell bodies (indicated by an arrow). Note the presence of presynaptic signal in the mushroom bodies, which is due to the background noise from the *trans-*Tango components and not the *Lk-GAL4*. **(C, E)** *Lkr* homozygous mutants show increased DILP2 immunoreactivity in insulin-producing cells (IPCs) of the adult brain. **(D, F)** Both *Lk* and *Lkr* homozygous mutants show increased DILP3 immunoreactivity in IPCs of the adult brain. (*** p < 0.001, **** p < 0.0001, as assessed by one-way ANOVA followed by Tukey’s multiple comparisons test). CTCF, corrected total cell fluorescence.

To establish the nature of connections (synaptic versus paracrine) between LK neurons and the IPCs, and to identify other neurons downstream of LK signaling, we employed the *trans*-Tango technique for anterograde trans-synaptic labeling of neurons [31]. Using two independent *Lk*-*GAL4* lines to drive expression of the system, we observed strong GFP labeling (presynaptic marker) in the SELK neurons for both lines (Figs 10A and 10B and S9) but presynaptic staining in the lateral horn region for only one line (Fig 10A and 10B). For both lines, expression of the postsynaptic marker (visualized by mtdTomato tagged with HA) was detected in several SEG neurons, some of which have axons that project to the pars intercerebralis (Fig 10A and 10B; S9 Fig). Since *Lkr* is expressed in the IPCs, which have dendrites in the tritocerebrum and subesophageal zone where the LK post-synaptic signal is found (S10 Fig), we asked whether the IPCs are postsynaptic to SELKs. However, no colocalization could be seen between the IPCs and postsynaptic signal of LKs (S9 Fig). In addition, the post-synaptic signal is not coexpressed with Hugin neurons (labeled with anti-CAPA antibody) although these have similar axonal projections (S11 Fig). Hence, these anatomical data indicate that the IPCs express the Lk receptor, but may receive non-synaptic (paracrine) inputs from LK neurons, or possibly LK signal via the circulation from the neurosecretory ABLKs.

Since *Lkr* is expressed in the IPCs, we asked whether the expression of DILPs is altered in *Lk* and *Lkr* mutants. In *Lk* mutant flies, DILP3 immunolabeling is increased, and in *Lkr* mutants both DILP2 and DILP3 levels are significantly higher (Fig 10C–10F), indicating that LK could affect the release of DILP2 and DILP3 (as increased immunolabeling has been proposed to reflect decreased peptide release [32]). No effect on DILP5 levels was seen for any of the mutants, suggesting that LK selectively modulates DILP function (S12 Fig).

**Fig 11:**
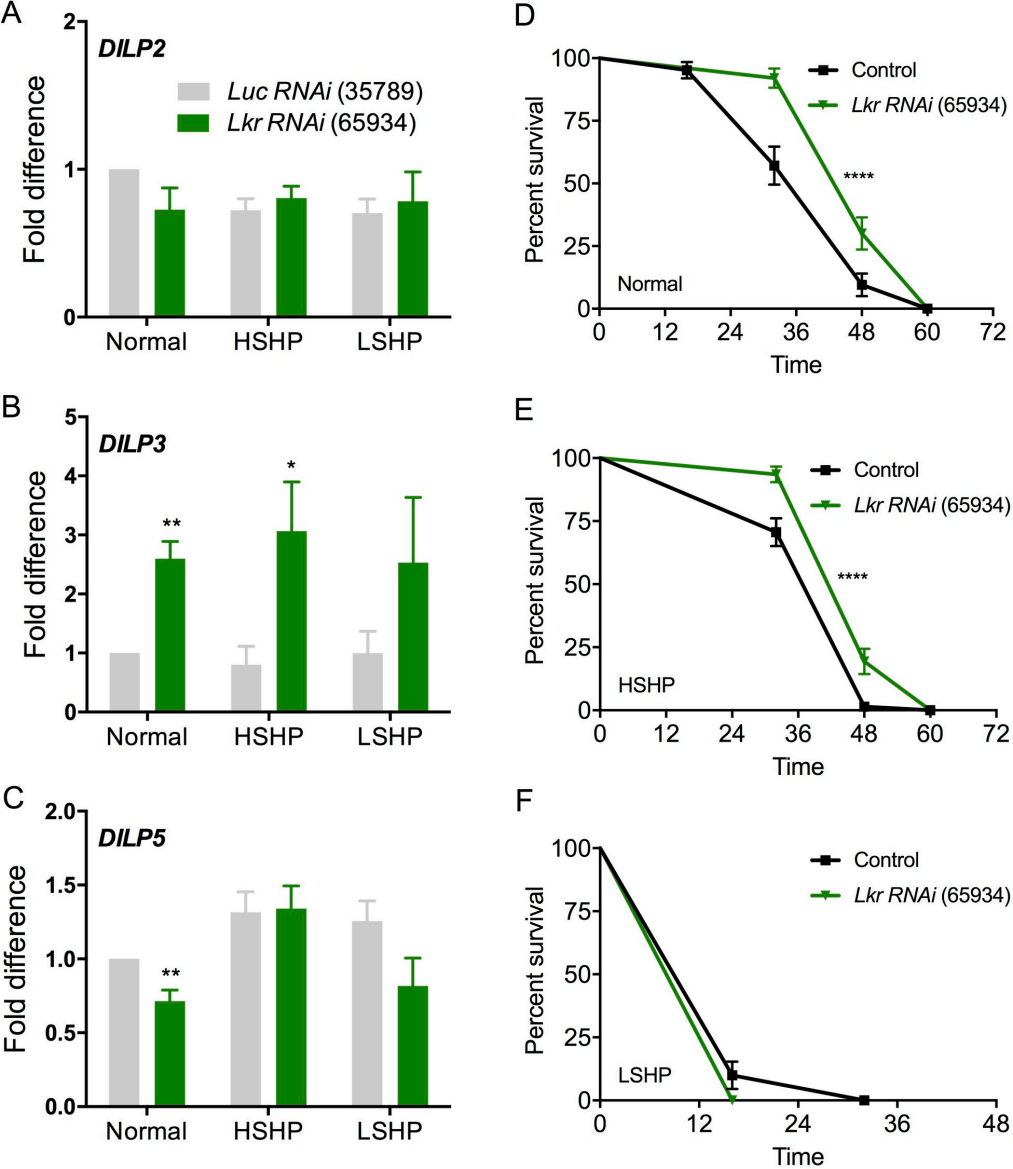
*Lkr* knockdown in insulin-producing cells affects insulin expression and starvation resistance. **(A)** Quantitative PCR shows no difference in *DILP2* transcript levels between control flies (*DILP2>Luciferase*) and flies with *Lkr* knockdown in insulin-producing cells (IPCs) that were reared as adults on normal diet, high sugar and high protein (HSHP) diet, or low sugar and high protein (LSHP) diet. **(B)** *DILP3* transcript levels are upregulated in *DILP2>Lkr-RNAi-#2 (BL#65934)* flies reared on normal and HSHP diets. **(C)** *DILP5* transcription is downregulated in *DILP2>Lkr-RNAi-#2 (BL#65934)* flies reared on normal diet. (* p < 0.05 and ** p < 0.01 as assessed by unpaired *t* test). Flies maintained as adults on **(D)** normal diet and **(E)** HSHP diet show increased starvation resistance whereas flies maintained on **(F)** LSHP diet have similar survival under starvation compared to control flies. For graphs D-F, data are presented in survival curves and the error bars represent standard error (**** p < 0.0001, as assessed by Log-rank (Mantel-Cox) test).

Next, we examined *DILP2*, *DILP3*, and *DILP5* transcript levels by qPCR after targeted knockdown of the *Lkr* in the IPCs of flies using two different *Lkr*-RNAi lines and a *DILP2*-*GAL4* driver. Also, different diets were tested since *DILP* expression in IPCs is influenced by carbohydrate and protein levels in the food [33]. The experimental flies developed to pupation on normal diet and were transferred as adults to three different diets: high sugar+high protein, low sugar+high protein, and normal diet. Knockdown of *Lkr* with *UAS-Lkr-RNAi-#1* in IPCs had no effect on *DILP* transcripts and starvation survival (S13 Fig), probably due to inefficient knockdown of *Lkr* with this construct. On the other hand, IPC-specific knockdown of *Lkr* with *UAS-Lkr-RNAi-#*2 (referred to as *Lkr-RNAi* from here on) impacted *DILP* transcripts and starvation survival in a diet-specific manner. Significant effects on *DILP* transcripts were only seen for *DILP3*, which was increased in flies after *Lkr*-*RNAi* under normal and high-sugar+high-protein diets, and *DILP5*, which was decreased in normal diet (Fig 11A–11C). Moreover, there was an increase in survival during starvation with reduced *Lkr* in IPCs in adult flies that had been maintained on normal and high sugar-high protein diets (Fig 11D–11F).

**Fig 12:**
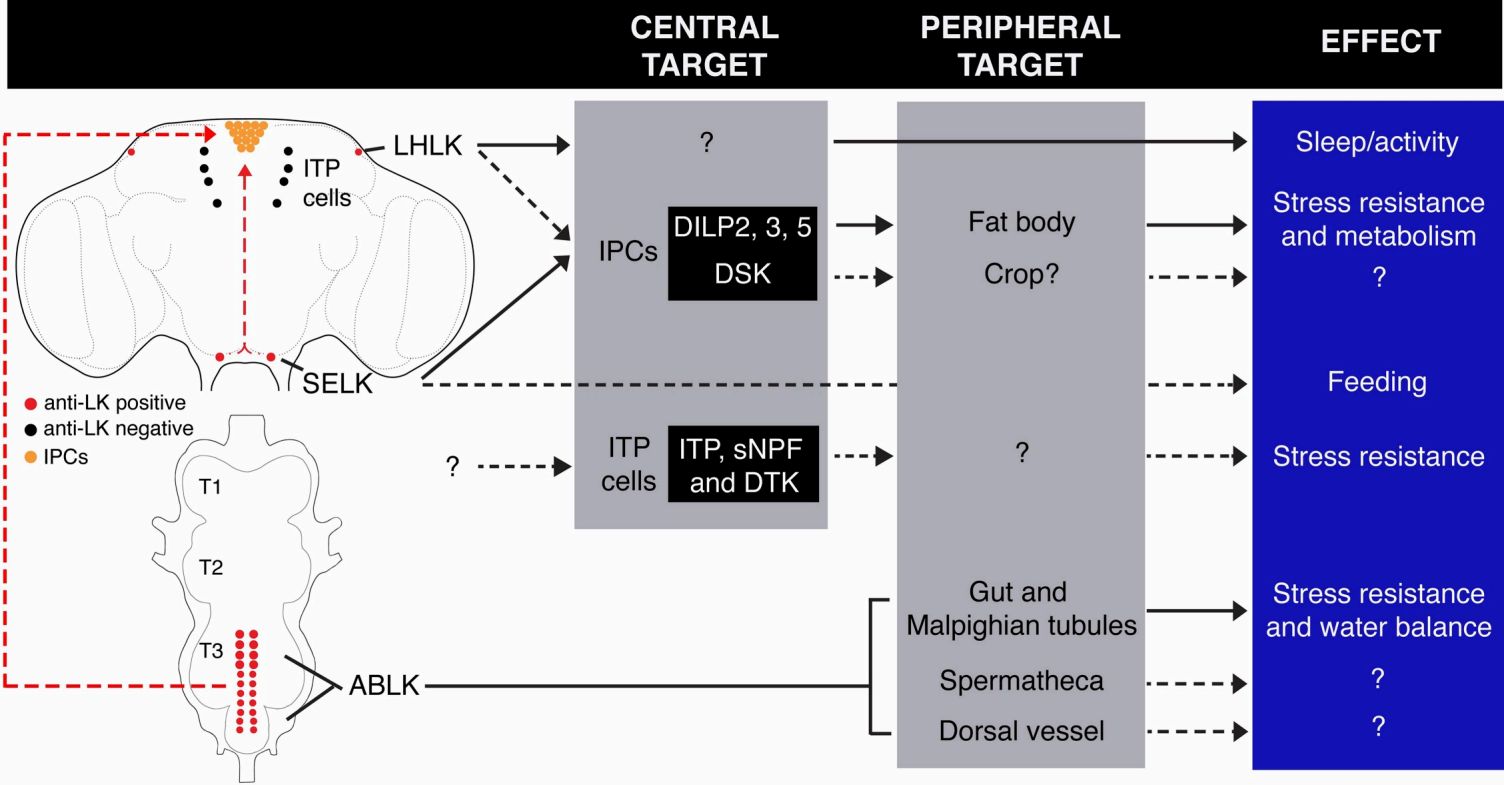
*Lk* signaling scheme. LK signaling scheme showing the location of all LK neurons, identified neurons downstream of LK neurons, target tissues, based on Lkr distribution and effects of LK signaling. Dashed arrows indicate probable links that need to be functionally validated. DSK, drosulfakinin; sNPF, short neuropeptide F; DTK, tachykinin.

Taken together, we identify roles for the signaling pathway comprising LK and its receptor within the CNS and that uniquely regulate physiological homeostasis. The *Lkr* expression in the periphery suggests that LK signaling is associated with water balance, gut function, and chemosensation (Fig 12). Within the CNS, LK signaling modulates specific neurosecretory cells of the brain that are known to regulate stress responses, feeding, metabolism, energy storage, and activity patterns, including sleep (Fig 12) [25,34–38].

## Discussion

In this study, we defined a set of effects caused by loss of LK signaling, which indicates that this neuropeptide homeostatically regulates physiology related to feeding, water homeostasis and metabolism, as well as associated stress, locomotor activity and metabolic rate. We suggest that LK regulates post-feeding physiology, metabolism, and behavior, as this seems to link most of the observed phenotypes observed after peptide and receptor knockdown. In S2 Table, we summarize effects of genetic manipulations of LK signaling from this study and earlier work and in Fig 12, we propose a scheme of functions for the different LK-expressing neurons both in the CNS and in the periphery. Our model suggests that LK acts on peripheral targets such as the intestine and renal tubules, and via intermediate neuroendocrine cells in the brain, such as the IPCs and ITP-producing neurons, which in turn act on peripheral targets such as the fat body, crop, intestine, and others that are yet to be determined.

In support of the physiological roles of LK signaling, we show distribution of *Lkr* expression in cells of the renal tubules and intestine, including the water-regulating rectal pads, as well as in the IPCs, which are known to signal with DILPs to affect feeding, metabolism, sleep, activity, and stress responses [34–37,39]. *Lkr* is also expressed by another set of brain neurosecretory cells (ipc-1/ipc-2a) known to regulate stress responses by means of three different coexpressed neuropeptides [25].

In the CNS of the adult fly, LK is produced at high levels by a small number of neurons of three major types: two pairs of interneurons in the brain (SELK and LHLK) and about 20 neurosecretory cells, ABLKs, in the abdominal ganglia [7,8]. Our data, taken together with earlier investigations (see S2 Table), enable us to propose that each of the three types of LK neurons plays a different functional role by acting on distinct targets. However, they appear to act cooperatively to regulate post-feeding physiology and behavior. There is mounting evidence that the ABLKs use LK as a hormonal signal that targets peripheral tissues, including the renal tubules [10], and that the brain LK neurons act in neuronal circuits within the CNS [11–13,40]. More specifically, the LHLK brain neurons are part of the output circuitry of the circadian clock in regulation of locomotor activity and sleep suppression induced by starvation [11,12,40], and the SELKs of the subesophageal zone may regulate feeding [13]. In fact we show here that these SELKs have axons that exit through subesophageal nerves known to innervate muscles of the feeding apparatus. We found in this study that the ABLKs display increased calcium activity in response to drinking in desiccated flies, but not during starvation, desiccation, or regular feeding. This finding further supports a role for the ABLKs and hormonal LK in regulation of water balance. These neurons have also been implicated more broadly in control of water and ion homeostasis and in responses to starvation, desiccation, and ionic stress [10]. The LHLKs and SELKs did not display changes in calcium signaling under the tested conditions, emphasizing the unique function of ABLKs in diuresis (see also [10]) and aligning with earlier work suggesting that the brain neurons play roles in activity/sleep and feeding [11,13,40].

The regulation of metabolic rate, as determined by measurement of CO_2_ production, is a novel phenotype that we can link to LK signaling. This may be associated with the overall activity of the flies, as suggested by the correlation between activity and CO_2_ levels in our data. Thus, the regulation of activity and metabolic rate might be coordinated by means of the LK neurons.

Using anatomical and experimental strategies, we identified a novel circuit linking LK to insulin signaling. *Lkr* expression was detected in the brain IPCs using two independently generated *GAL4* lines plus single-cell transcriptome analysis. We also observed that *Lk* and *Lkr* mutants displayed increased levels of DILP2 and DILP3 immunoreactivity in the brain IPCs, and targeted knockdown of *Lkr* in IPCs increased *DILP3* expression. Associated with this we found that *Lkr*-RNAi targeted to IPCs increased resistance to starvation. However, using the *trans*-Tango method for anterograde trans-synaptic labeling [31], we could not demonstrate direct synaptic inputs to the IPCs from LK neurons. We found that LHLK neuronal processes do not overlap with those of IPCs in the brain. The SELKs drove postsynaptic marker signal in sets of neurons in the SEG, some of which have processes impinging on the IPCs. These findings suggest that LHLKs and SELKs form no conventional synaptic contacts with IPCs, but paracrine LK signaling to these neurons cannot be excluded since the SELK neurons have processes in close proximity to IPCs in the tritocerebrum and the subesophageal zone. Non-synaptic paracrine signaling with neuropeptides has been well established in mammals (see [41–43]) and is likely to occur also in insects [44]. Alternatively, the LK input to IPCs could occur systemically at the peripheral axon terminations of the IPCs after hormonal release from ABLKs. Whether acting in a paracrine or a hormonal fashion, LK appears to regulate the IPCs at the level of transcription and release of DILPs. Thus, some phenotypes seen after the global knockdown of LK and its receptor are likely to arise via secondary effects of insulin signaling. This suggests another layer of regulatory control whereby LK-driven modulation of DILP production and release could affect metabolism, stress responses, and longevity [reviewed by [39,45,46]]. Our findings, therefore, add LK as yet another regulator of the *Drosophila* IPCs, which have previously been shown to be under the influence of several other neuropeptides and neurotransmitters [reviewed in [39,45]]. It is noteworthy that at the levels of both transcription and presumed release the effect of LK on IPCs is selective, affecting DILP2, DILP3, and *DILP3* only.

We suggest that LK signaling may be nutrient-dependent and regulates post-feeding physiology and behavior, that can be observed in the mutants as reduced metabolic rate and locomotor activity, diminished PER, and reduced diuresis, as well as increased resistance to starvation and desiccation. Our data also indicate that in wild type flies, LK triggers release of IPC-derived DILPs that are required for post-feeding metabolism and satiety, and it acts on other cells to induce diuresis, and to increase activity (especially evening activity) and metabolic rate. An orchestrating role of LK signaling requires that the three types of LK neurons communicate with each other or are under simultaneous control by common sets of regulatory neurons. Alternatively, all the LK neurons could possess endogenous nutrient-sensing capacity whereby they can monitor levels of amino acids or carbohydrates in the organism. There is evidence for nutrient sensing in LHLK neurons [47]. This has also been shown for the brain neurosecretory cells expressing DH44, DILP and corazonin [32,48–50]. Of the LK neurons, only the ABLKs and SELKs exhibit overlapping processes that could support direct communication, so it is more likely that other neurons form the link between these three sets of neuroendocrine cells. Such neurons are yet to be identified, but it has been shown that all the LK neurons express the insulin receptor, dInR [19,22]. This may suggest that the LK neurons could receive nutrient-related information from insulin-producing cells in the brain or elsewhere.

In conclusion, we found that LK signaling is likely to modulate postprandial physiology and behavior in *Drosophila*. Food ingestion is followed by increased insulin signaling, activation of diuresis, increased metabolic rate, and lowered locomotor activity and increased sleep [12,15,32,45]. Flies mutated in the *Lk* and *Lkr* genes display phenotypes consistent with a role in regulation of insulin signaling, metabolic stress responses, diuresis, metabolic rate, and locomotor activity, all part of postprandial physiology.

## Methods

### Fly lines and husbandry

All fly strains used in this study (**Table 1**) were reared and maintained at 25°C on enriched medium containing 100 g/L sucrose, 50 g/L yeast, 12 g/L agar, 3 ml/L propionic acid, and 3 g/L nipagin, unless otherwise indicated. Experimental flies were reared under normal photoperiod (12 hours light: 12 hours dark; 12L:12D). Adult males 6–8 days post-eclosion were used for behavioral experiments. For some imaging experiments, females of the same age were also utilized. For *trans-*Tango analysis, flies were reared at 18°C, and adult males 2–3 weeks old post-eclosion were used.

**Table 1:** Fly strains used in this study.

Fly strain	Inserted on chromosome	Source / reference
*w*^*1118*^ (RNAi control)	-	BDSC
*w*^*1118*^ (mutant control)	-	BDSC #5905 [51]
*w*^*1118*^*; Lk-GAL4* ^*CC9*^ (mutant)		This study
*w*^*1118*^*; Lkr-GAL4* ^*CC9*^ (mutant)		This study
*w*^*1118*^*; Lk-GAL4*	II	BDSC #51993, (K. Asahina and D. Anderson). [52]
*w*^*1118*^*; Lk-GAL4*	III	Y. J. Kim [53]
*w*^*1118*^*; Lk-GAL4*	II	P. Herrero [8]
*w*^*1118*^*; Lkr-GAL4*::*p65* (*Lkr-GAL4*)	III	This study
*w; DILP2-GAL4*	III	E. Rulifson [35]
*yw; Sco/CyO; UAS-mCD8;;GFP*	III	BDSC
*JFRC81-10xUAS-IVS-Syn21-GFP-p10*		[54]
*JFRC29-10xUAS-IVS-myr*::*GFP-p10*		[54]
*UAS-DenMark*		BDSC #33064, (donated by C. Wegener). [55]
*UAS-Dscam-GFP*		Tzumin Lee, (donated by C. Wegener). [56]
*UAS-trans-Tango*	X and II	BDSC #77124 [31]
*UAS-IMP-TNT* (inactive control)	II	BDSC #28840
*UAS-TNT*	X	BDSC #28996
*UAS-CaLexA*	II and III	BDSC #66542 [23]
*w*^*1118*^*; UAS-Lkr*		B. Al-Anzi [13]
*w*^*1118*^*; UAS-Lk*	II	This study
*UAS-Lkr-RNAi-#1* (JF01956)	III	BDSC #25936
*UAS-Lkr-RNAi-#2* (HMC06205)	III	BDSC #65934
*UAS-Luciferase* (pValium TRiP RNAi vector control)	III	BDSC #35789

For *DILP2>Lkr-RNAi* qPCR, crosses were established in normal food (NutriFly Bloomington formulation) and eggs were laid for 24 hours. After adult eclosion, males were transferred to alternative diets (normal diet described above; high-sugar high-protein: normal diet except with 20% sucrose and 10% yeast; low-sugar high-protein: normal diet except 5% sucrose and 10% yeast). After 5–7 days on these media, heads were dissected for qPCR, and other animals were transferred to starvation vials containing 1% agarose in water.

### Generation of *GAL4* knock-in mutants and transgenic lines

*Lk-/-* and *Lkr -/-* were generated using the CRISPR/Cas9 system to induce homology-dependent repair (HDR) using one guide RNA (*Lk-/-*: GATCTTTGCCATCTTCTCCAG and *Lkr-/-*: GTAGTGCAATACATCTTCAG). At gRNA target sites, a donor plasmid was inserted containing a *GAL4*::*VP16* and floxxed 3xP3-RFP cassette. For *Lk-/-*, the knock-in cassette was incorporated immediately following the *ATG* translational start site (+4bp to +10bp, relative to start site). For *Lkr-/-*, the knock-in cassette was incorporated upstream of the *ATG* (-111bp to -106bp, relative to start site). All mutations were generated in the *w*^*1118*^ background. Proper insertion loci for both mutations were validated by genomic PCR. CRISPR gene editing was done by WellGenetics (Taipei City, Taiwan).

To prepare the *Lkr-GAL4*::*p65* line, recombineering approaches based on previous methods [57] were used: in brief: a large genomic BAC with *GAL4*::*p65* replacing the first coding region of *Lkr*, thereby retaining regulatory flanks and introns). First, a landing-site cassette was prepared: *GAL4* and terminator homology arms were amplified from *pBPGUw* [58] and added to the flanks of the marker *RpsL-kana* [59], which confers resistance to kanamycin and sensitivity to streptomycin. *Lkr*-specific arms were added to this landing-site cassette by PCR with the following primers, made up of 50 bases of *Lkr*-specific homology (lower case) plus regions matching the GAL4/terminator sequences:

Lkr-F: tcatatcctcattaggatacacaactaaaactaaaaaacgaaaaagtgttATGAAGCTACTGTCTTCTATCGAACAAGC

Lkr-R: tggatgagtcgcgtccccagttgcttgaagggattagagagtatacttacGATCTAAACGAGTTTTTAAGCAAACTCACTCCC

Note the underlined *ATG*, reflecting the integration of *GAL4* at the *Lkr* initiation site. The PCR product was recombined into bacterial artificial chromosome CH321-16C22 [60] (obtained from Children’s Hospital Oakland Research Institute, Oakland, CA, USA), which contains the *Lkr* locus within 90 kb of genomic flanks. Recombinants were selected on kanamycin. Next, this landing pad was replaced by full-length GAL4::p65+terminators amplified from *pBPGAL4*.*2*::*p65Uw* [61], and recombinants were screened for streptomycin resistance. Recombination accuracy was confirmed by sequencing, and the construct was integrated into *attP40* by Rainbow Transgenic Flies (Camarillo, CA, USA).

### RT-qPCR

To quantify *Lk* and *Lkr* transcript levels in mutant flies, the following method was used. Briefly, ten or more fed flies were flash frozen for each sample. Total RNA was extracted from whole flies using RNeasy Tissue Mini kit (Qiagen) according to the manufacturer’s protocol. RNA samples were reverse transcribed using iScript (Biorad), and the subsequent cDNA was used for real-time RT-qPCR (Biorad CFX96, SsoAdvanced Universal SYBR Green Supermix qPCR Mastermix Plus for SYBRGreen I) using 1.7 ng of cDNA template per well and a primer concentration of approximately 300 nM. The primers used are listed in **Table 2**. Triplicate measurements were conducted for each sample.

**Table 2:** Primers used for qPCR.

Primer	Sequence (5’ to 3’)
Primers for *Lk* and *Lkr* transcripts	
*Lk* forward	GCCTTTGGCCGTCAAGTCTA
*Lk* reverse	TGAACCTGCGGTACTTGGAG
*Lkr* forward	GGAGGAAGCAGAATTTGAGCG
*Lkr* reverse	AAAGTGTTGCCAATGACGGC
*Actin5C* forward	AGCGCGGTTACTCTTTCACCAC
*Actin5C* reverse	GTGGCCATCTCCTGCTCAAAGT
*β-tubulin* forward	GCAGTTCACCGCTATGTTCA
*β-tubulin* reverse	CGGACACCAGATCGTTCAT
Primers for *DILP2*, *3* and *5* transcripts	
*DILP2* forward	CTCAACGAGGTGCTGAGTATG
*DILP2* reverse	GAGTTATCCTCCTCCTCGAACT
*DILP3* forward	CAACGCAATGACCAAGAGAAC
*DILP3* reverse	GCATCTGAACCGAACTATCACTC
*DILP5* forward	ATGGACATGCTGAGGGTTG
*DILP5* reverse	GTGGTGAGATTCGGAGCTATC
*RpL32/Rp49* forward	AGTATCTGATGCCCAACATCG
*RpL32/Rp49* reverse	CAATCTCCTTGCGCTTCTTG

To quantify *DILP2*, *3* and *5* transcript levels following *DILP2>Lk-RNAi*, the following method was used. *DILP2-GAL4* and *UAS-RNAi* animals (*Lkr-RNAi-#1* and *-#2*, plus a matched *UAS-Luciferase* as a control for effects of genetic background) were mated and allowed to lay eggs for 24 hours in vials containing normal food; adult males from these crosses were then transferred to vials of normal food or high-sugar, high-protein or low-sugar high-protein diet. After 7 days, heads were dissected on ice into extraction buffer, and RNA was extracted with the Qiagen RNeasy Mini kit (#74106) with RNase-free DNase treatment (Qiagen #79254). cDNA was prepared using the High-Capacity cDNA Reverse Transcription Kit with RNase Inhibitor (ThermoFisher #4268814), and qPCR was performed using the QuantiTect SYBR Green PCR Kit (Fisher Scientific #204145) and an Mx3005P qPCR system (Agilent Technologies). Expression levels were normalized against RpL32 (Rp49), whose levels have been determined to be stable under dietary modification [33,62]. The primers used are listed in Table 2. Samples were prepared in four biological replicates of 10 heads each, and each biological replicate was assayed in two technical replicates.

### Immunohistochemistry and imaging

Immunohistochemistry for *Drosophila* larval and adult tissues was performed as described earlier [10,63]. Briefly, tissues were dissected in phosphate-buffered saline (PBS) and fixed in 5% ice-cold paraformaldehyde (2 hours for larval samples and 3.5–4 hours for adults). Samples were then washed in PBS and incubated for 48 hours at 4°C in primary antibodies diluted in PBS with 0.5% Triton X-100 (PBST) (**Table 3).** Samples were thereafter washed with PBST and incubated for 48 hours at 4°C in secondary antibodies diluted in PBST (**Table 3**). Following this incubation, some samples (peripheral tissues) were incubated with rhodamine-phalloidin (1:1000; Invitrogen) and/or DAPI as a nuclear stain (1:1000; Sigma) diluted in PBST for 1 hour at room temperature. Finally, all samples were washed with PBST and PBS, and then mounted in 80% glycerol. An alternative procedure was used for the adult gut to prevent tissues from rupturing. Briefly, intestinal tissues (proventriculus, crop, midgut, hindgut, and MTs) were fixed at room temperature for 2 hours, washed in PBS, incubated in rhodamine-phalloidin for 1 hour and washed in PBST and then PBS before mounting. Samples were imaged with a Zeiss LSM 780 confocal microscope (Jena, Germany) using 10X, 20X, or 40X oil immersion objectives. Images for the whole fly, proboscis, and wing were captured using a Zeiss Axioplan 2 microscope after quickly freezing the fly at -80°C. Cell fluorescence was measured as described previously [10]. Confocal and fluorescence microscope images were processed with Fiji [64] for projection of z-stacks, adjustment of contrast and brightness, and calculation of immunofluorescence levels.

**Table 3:** Antibodies used for immunohistochemistry.

Antibody	Antigen	Source / reference	Dilution
***Primary antisera***			
Rabbit anti-LK	*Leucophaea maderae* leucokinin I	Own production [65]	1:2000
Rabbit anti-DromeLkr	*Drosophila* Lkr C-terminus (GIYNGSSGQNNNVN)	[14]	1:1000
Guinea pig anti-ITP	*Drosophila* ITP (amidated)	(H. Dircksen and D. R. Nässel, unpublished)	1:4000
Rabbit anti-DILP2	*Drosophila* DILP2	From J. A. Veenstra [66]	1:2000
Rabbit anti-DILP3	*Drosophila* DILP3	From J. A. Veenstra [66]	1:2000
Rabbit anti-DILP5	*Drosophila* DILP5	Own production [67]	1:2000
Rabbit anti-CAPA	*Periplaneta americana* CAPA-PVK-2	R. Predel [68]	1:4000
Mouse anti-GFP	Jellyfish GFP	Invitrogen	1:1000
Chicken anti-GFP	Jellyfish GFP	Invitrogen	1:1000
Mouse anti-HA	HA-tag (YPYDVPDYA)	Invitrogen	1:1000
***Secondary antisera***			
Goat anti-mouse Alexa Fluor 488	-	Invitrogen	1:1000
Goat anti-rabbit Alexa Fluor 546	-	Invitrogen	1:1000
Goat anti-guinea pig Cyanine3	-	Invitrogen	1:500
Goat anti-rabbit Cyanine5	-	Life Technologies	1:500
Goat anti-chicken Alexa Fluor 488		Life Technologies	1:1000
Goat anti-mouse Alexa Fluor 546	-	Life Technologies	1:1000
***Other fluorophores***			
Rhodamine-phalloidin	-	Invitrogen	1:1000
DAPI	-	Sigma	1:1000

### Calcium activity in LK neurons

Calcium activity of LK neurons following various stresses was measured using the CaLexA (Calcium-dependent nuclear import of LexA) technique [23]. Briefly, the CaLexA sensor was expressed in LK neurons using the *Lk-GAL4*. Next, 6-8-day-old males were transferred to a vial containing either nothing (desiccation), aqueous 1% agar (starvation) or artificial diet (normal food) and incubated for 16 hours. In addition, one set of flies were desiccated for 13 hours and then transferred to a vial containing 1% agar (re-watered). Following this period, the flies were fixed, dissected brains were processed for immunohistochemistry, and the GFP fluorescence was quantified as described above.

### Stress-resistance assays

To assay for survival under desiccation (dry starvation) and starvation, flies were kept in empty vials or vials containing 5 ml of 0.5% aqueous agarose (A2929, Sigma-Aldrich), respectively. Four biological replicates and 3 technical replicates for each biological replicate were performed for each experiment. For each technical replicate, 15 flies were kept in a vial and their survival was recorded every 3 to 6 hours until all the flies were dead. The vials were placed in incubators at 25°C under normal photoperiod conditions (12L:12D).

### Water-content measurements

For water-content measurements, 15 flies per replicate (4 biological replicates) were either frozen immediately on dry ice or desiccated as above for 9 hours and then frozen. The samples were stored at -80°C until use. To determine their wet weight, flies were brought to room temperature and their weight was recorded using a Mettler Toledo MT5 microbalance (Columbus, Ohio, USA). The flies were then dried for 24–48 hours at 60°C before their dry weight was recorded. The water content of the flies was determined by subtracting dry weight from wet weight.

### Capillary feeding assay

Long-term food intake of individual flies was quantified using a modified capillary feeding (CAFE) assay [19,69]. Capillaries were loaded with food comprising 5% sucrose, 2% yeast extract, and 0.1% propionic acid. Food consumption was measured daily, and the cumulative food intake over 3 days was calculated. The experiment consisted of 4 biological replicates and 10 flies per replicate for each genotype.

### Blue dye feeding assay

Short-term food intake was measured as previously described [70]. Briefly, flies were starved for 24 hours on 1% agar (Fisher Scientific) or maintained on standard fly food. At ZT0, flies were transferred to food vials containing 1% agar, 5% sucrose, and 2.5% blue dye (FD&C Blue Dye No. 1, Spectrum). Following 30 minutes of feeding, flies were flash frozen on dry ice, and four flies per sample were homogenized in 400 μL PBS (pH 7.4, Fisher Scientific). Color spectrophotometry was used to measure absorbance at 655 nm in a 96-well plate reader (Millipore, iMark, Bio-Rad). Baseline absorbance was determined by subtracting the absorbance measured in non-dye fed flies from each experimental sample.

### Proboscis extension reflex

Flies were collected and placed on fresh food for 24 hours, then starved for 24 hours in vials containing 1% agar. Flies were then anaesthetized under CO_2_, and their thorax and wings were glued with nail polish to a microscopy slide, leaving heads and legs unconstrained. Following 1-hour recovery in a humidified chamber, the slide was mounted vertically under the dissecting microscope (SM-3TX-54S, AmScope) and proboscis extension reflex (PER) was observed. PER induction was performed as described previously [71]. Briefly, flies were satiated with water before and during experiments. Flies that did not water-satiate within 5 minutes were excluded from the experiment. A 1-ml syringe (Tuberculin, BD&C) with an attached pipette tip was used for tastant (sucrose) presentation. Tastant was manually applied to tarsi for 2–3 seconds 3 times with 10-second inter-trial intervals, and the number of full proboscis extensions was recorded. Tarsi were then washed with distilled water between applications of different concentrations of sucrose (0.1, 1.0, 10, and 100 mM), and flies were allowed to drink water during the experiment *ad libitum*. Each fly was assayed for response to tastants. PER response was calculated as a percentage of proboscis extensions to total number of tastant stimulations to tarsi.

### Activity and metabolic rate

Activity and metabolic rate (MR) was simultaneously recorded using the setup described earlier [24]. Briefly, MR was measured at 25°C through indirect calorimetry, measuring CO_2_ production of individual flies with a CO_2_ analyzer (LI-7000, LI-COR). Baseline CO_2_ levels were measured from an empty chamber, alongside five behavioral chambers, each measuring the CO_2_ production of a single male fly. The weight of a group of 10 flies was used to normalize metabolic rate since *Lk* mutants weighed significantly more than control *w*^*1118*^ flies. Flies were anesthetized using CO_2_ for sorting and allowed 24 hours acclimation before the start of an experiment. Flies were placed in glass tubes that fit a custom-built *Drosophila* Locomotor Activity Monitor (Trikinetics, Waltham, MA), containing a single food tube containing 1% agar plus 5% sucrose with green food coloring (McCormick). Locomotor activity data was calculated by extracting 10-minute activity periods for 24 hours using a custom generated Python program. CO_2_ output was measured by flushing air from each chamber for 75 seconds, providing readout of CO_2_ accumulation over the 10-minute period. This allowed for the coordinated and simultaneous recordings of locomotor activity and metabolic rate.

### Locomotor activity

*Drosophila* activity monitoring system (DAMS; Trikinetics, Waltham, MA) detects activity by monitoring infrared beam crossings for each animal. These data were used to calculate locomotor activity using the *Drosophila* Sleep Counting Macro [72]. Flies were anaesthetized under CO_2_ and loaded into DAMS tubes containing standard fly food for acclimation. After 24 hours acclimation in DAMS tubes with food, baseline activity was measured for 24 hours. Tubes were maintained in a 25°C incubator with 12:12 LD cycles.

### Mining public datasets for expression of genes

*Lkr* distribution in various tissues was determined by mining the FlyAtlas database [28]. *Lkr* expression in the different regions of the gut and its cell types was obtained using Flygut-*seq* [29]. A single-cell transcriptome atlas of the *Drosophila* brain was mined using SCope (http://scope.aertslab.org) to identify genes coexpressed with *Lkr* [30].

### Statistical analyses

In all bar graphs, the data are presented as means ± s.e.m. In all box-and-whisker plots, each individual value has been plotted and the horizontal line represents the median. Unless stated otherwise, one-way analysis of variance (ANOVA) followed by Tukey’s multiple comparisons test was used for comparisons between three genotypes and an unpaired *t* test was used for comparisons between two genotypes. All statistical analyses were performed using GraphPad Prism with a 95% confidence limit (*p* < 0.05). Survival and stress curves were compared using Mantel–Cox log-rank test. All data sets are available in the S1 Data File.

## Supporting information

S1 Data File Raw data files for all graphs. (XLSX)

S1 Table p-values for the proboscis extension reflex data in Fig 5. p-values below 0.05 have been highlighted in grey. Wilcoxon Rank-Sum was used for comparison between two genotypes, while Kruskal-Wallis with Steel-Dwass post-hoc test was used for two or more genotypes. These tests were performed at each concentration independently.
(PDF)

S2 Table Summary of genetic manipulations that demonstrate functional roles of LK signaling in this study and in published work. (PDF)

S1 Fig Total activity (measured using DAMS) of *Lk* and *Lkr* mutants. Total locomotor activity of single flies measured over 24 hours is lowered for homozygous and heterozygous **(A)** *Lk* and **(B)** *Lkr* mutants. The activity was monitored using a standard *Drosophila* Activity Monitor (DAMS). (*** p < 0.001, **** p < 0.0001, as assessed by one-way ANOVA).
(JPG)

S2 Fig The *Lk-GAL4*^*CC9*^ drives GFP expression in the adult CNS. *Lk-GAL4*^*CC9*^ drives GFP (*pJFRC81-10xUAS-Syn21-myr*::*GFP-p10*) expression in the adult **(A)** brain and **(B)** ventral nerve cord (VNC). SELK, subesophageal LK neurons; ABLK, abdominal LK neurons. *Lk-GAL4*^*CC9*^ also drives GFP expression in four pairs of neurons in the brain (indicated by the white box). **(C)** These four pairs of neurons display very weak LK-immunoreactivity and are positive for ion transport peptide-immunoreactivity. GFP expression also colocalizes with anti-LK staining in the SELKs and lateral horn LK neurons (LHLK). **(D)** *Lk-GAL4*^*CC9*^ drives GFP expression in ABLKs (labeled with anti-LK antiserum) in the VNC.
(JPG)

S3 Fig *Lk-GAL4*^*CC9*^ and *Lkr-GAL4*^*CC9*^ drive GFP expression in the larval CNS. **(A)** *Lk-GAL4*^*CC9*^ drives GFP (*pJFRC81-10xUAS-Syn21-myr*::*GFP-p10*) expression in neurosecretory cells in the larval brain and ventral nerve cord (NVC). **(B)** *Lkr-GAL4*^*CC9*^ drives GFP (*UAS-mCD8;;GFP*) expression in larval CNS. Note the GFP expression in motor neurons in the VNC.
(JPG)

S4 Fig The *Lkr-GAL4*^*CC9*^ drives GFP expression in adult peripheral tissues. *Lkr-GAL4*^*CC9*^ drives GFP (*pJFRC81-10xUAS-Syn21-myr*::*GFP-p10*) expression in the adult **(A)** dorsal vessel and peripheral neurons (indicated by an arrow), **(B)** legs, **(C)** proboscis, and **(D)** wings. Note the expression of *Lkr* in nerve fibers closely associated with the anti-LK immunostaining in **(A)**.
(JPG)

S5 Fig The *Lkr-GAL4*^*CC9*^ drives GFP expression in larval gut and Malpighian tubules. *Lkr-GAL4*^*CC9*^ drives GFP (*pJFRC81-10xUAS-Syn21-myr*::*GFP-p10*) expression in the larval **(A)** gut, **(B)** gastric caeca and anterior midgut, **(C)** midgut, and **(D)** anti-DromeLkr-expressing stellate cells in Malpighian tubules. Nuclei in all the preparations have been stained with DAPI (blue).
(JPG)

S6 Fig The *Lkr-GAL4* drives GFP expression in gut and Malpighian tubules. *Lkr-GAL4* drives GFP (*pJFRC29-10xUAS-myr*::*GFP-p10*) expression in **(A)** the larval stellate cells of Malpighian tubules, **(B)** larval hindgut, and **(C-E)** adult stellate cells (labeled with anti-DromeLkr antiserum). Note that the adult stellate cells can be (**C**) cuboidal or (**D**) star-shaped (indicated by an arrow).
(JPG)

S7 Fig *Lkr-GAL4* drives GFP (*UAS-mCD8;;GFP*) expression in larval and adult CNS. **(A)** *Lkr-GAL4* drives GFP expression in several neurons of the larval CNS, including a pair of abdominal Lk neurons stained with anti-Lk antiserum (indicated by arrow). In adults, *Lkr-GAL4* drives GFP expression in **(B)** T1 and T2 thoracic neuromeres and **(C)** T3 thoracic neuromere.
(JPG)

S8 Fig The *Lkr-GAL4*^*CC9*^ drives GFP expression in the adult CNS. *Lkr-GAL4*^*CC9*^ drives GFP (*UAS-mCD8;;GFP*) expression in **(A)** the brain and **(B)** ventral nerve cord. The inset in **(A)** represents a smaller Z-stack, which shows GFP expression in the fan-shaped body. These preparations were counterstained with anti-nc82 antiserum. **(C)** *Lkr-GAL4*^*CC9*^ drives GFP (*pJFRC81-10xUAS-Syn21-myr*::*GFP-p10*) expression in neurons of the abdominal ganglia that do not express LK.
(JPG)

S9 Fig Anatomical relations between LK and insulin signaling components. **(A)** Expression of *trans*-Tango components [31] using *Lk-GAL4* (from P. Herrero) generates a presynaptic signal (labeled with anti-GFP antibody) in the subesophageal ganglion (SEG) and a postsynaptic signal (labeled with anti-HA antibody) in the SEG and pars intercerebralis, which does not colocalize with insulin-producing cells or their axons (labeled with anti-DILP2 antibody). **(B)** Higher magnification of the SEG showing the presynaptic and postsynaptic signals and the lack of colocalization with anti-DILP2 staining.
(JPG)

S10 Fig The processes of IPCs in pars intercerebralis and tritocerebrum/ subesophageal zone have dendrite properties. Using dendrite-directed UAS constructs, fluorescent labeling can be seen in IPC processes in pars intercerebralis and tritocerebrum/subesophageal zone, shown in inverted images. **(A)** *DILP2*-*GAL4* driven *Dscam*-GFP and **(B)** *DILP2*-*GAL4* driven *DenMark*-RFP. These images were kindly provided by Dr. Yiting Liu.
(JPG)

S11 Fig Anatomical interactions between LK and CAPA/hugin signaling. **(A)** Expression of *trans*-Tango components [31] using *Lk-GAL4* generates a post-synaptic signal (labeled with anti-HA antibody) in the tritocerebrum and pars intercerebralis which does not colocalize with CAPA/hugin axons (labeled with anti-CAPA antibody). **(B)** Higher magnification of the subesophageal ganglion showing the pre-synaptic and post-synaptic signals and the lack of colocalization with anti-CAPA staining.
(JPG)

S12 Fig DILP5 levels are unaltered in *Lk* and *Lkr* mutants. **(A)** *Lk* and *Lkr* homozygous mutants do not display any difference in DILP5 immunoreactivity in insulin-producing cells (IPCs) of the adult brain. **(B)** Fluorescence intensity measurement of IPCs shows no difference in DILP5 immunoreactivity in *Lk* and *Lkr* mutant flies compared to control flies. CTCF, corrected total cell fluorescence.
(JPG)

S13 Fig Effect of *Lkr* knockdown in insulin-producing cells on insulin expression and starvation resistance. Quantitative PCR shows no difference in **(A)** *DILP2*, **(B)** *DILP3*, and **(C)** *DILP5* transcript levels between control flies (*DILP2>Luciferase*) and flies with *Lkr* knockdown in insulin-producing cells (IPCs) (*DILP2>Lkr-RNAi-#1 (BL#25936)* that were reared as adults on normal diet, high sugar and high protein (HSHP) diet or low sugar and high protein (LSHP) diet. Flies maintained as adults on **(E)** HSHP diet show increased starvation resistance whereas flies maintained on **(D)** normal diet and **(F)** LSHP diet have similar survival under starvation compared to control flies. For graphs D-F, data are presented in survival curves and the error bars represent standard error (*** p < 0.001, as assessed by Log-rank (Mantel-Cox) test).
(JPG)
