# Supplementary material for: Modulation of Drosophila post-feeding physiology and behavior by the neuropeptide leucokinin
Source: PLoS Genet. 2018 Nov 20;14(11):e1007767. doi: 10.1371/journal.pgen.1007767 (PMC6245514; doi:10.1371/journal.pgen.1007767)
Supplement: S1 Table — p-values below 0.05 have been highlighted in grey. Wilcoxon Rank-Sum was used for comparison between two genotypes, while Kruskal-Wallis with Steel-Dwass post-hoc test was used for two or more genotypes. These tests were performed at each concentration independently. (PDF) [file pgen.1007767.s002.pdf]

Supplementary Table 1: p-values for the proboscis extension reflex data in Figure 5. p-values below 0.05 have been highlighted in grey. Wilcoxon Rank-Sum was used for comparison between two genotypes, while Kruskal-Wallis with Steel-Dwass post-hoc test was used for two or more genotypes. These tests were performed at each concentration independently.

	**0.1mM**	**1mM**	**10mM**	**100mM**
**Figure 5A**				
w1118 vs Lk+/-	0.4716	0.4609	0.1857	< 0.0001
w1118 vs Lk-/-	0.9719	0.2162	0.0203	< 0.0001
Lk+/- vs Lk-/-	0.5125	0.0941	0.6813	0.5880
**Figure 5B**				
w1118 vs Lk-/-	0.1370	0.0649	< 0.0001	< 0.0001
UAS-Lk; Lk-/- vs Lk-/-	0.5713	0.5025	0.0113	0.0027
w1118 vs UAS-Lk; Lk-/-	0.9181	0.8094	0.9483	0.0045
**Figure 5C**				
Lk > TNT vs Lk > IMP TNT	0.0608	0.0549	0.0120	0.0295
**Figure 5D**				
w1118 vs UAS-Lkr; Lkr-/-	0.7218	0.9641	0.7593	0.4060
w1118 vs Lkr+/-	0.7780	0.8045	0.9101	0.3203
UAS-Lkr; Lkr-/- vs Lkr+/-	0.9871	0.7140	0.6810	0.0523
UAS-Lkr; Lkr-/- vs Lkr-/-	0.1947	0.0093	0.0026	< 0.0001
Lkr+/- vs Lkr-/-	0.0884	0.0040	0.0079	0.0966
w1118 vs Lkr-/-	0.1349	< 0.0001	< 0.0001	0.0008
