# Supplementary material for: Modulation of Drosophila post-feeding physiology and behavior by the neuropeptide leucokinin
Source: PLoS Genet. 2018 Nov 20;14(11):e1007767. doi: 10.1371/journal.pgen.1007767 (PMC6245514; doi:10.1371/journal.pgen.1007767)
Supplement: S2 Table — (PDF) [file pgen.1007767.s003.pdf]

Assay	Genotype	Phenotype	Reference
Starvation resistance	Lk and Lkr mutants<br>Lk > Lk RNAi<br>DILP2 > Lkr RNAi	Increased survival<br>Increased survival<br>Increased survival [a]	This study<br>[1]<br>This study
Desiccation resistance	Lk and Lkr mutants<br>Lk > Lk RNAi<br>Lk > TrpA1<br>Lk; $Gal80^{ts}$ > Ork	Increased survival<br>Increased survival<br>Decreased survival<br>Increased survival	This study<br>[1]<br>[2]<br>[2]
Water content	Lk and Lkr mutants<br>Lk > Lk RNAi	Increased<br>Increased	This study<br>[1]
Proboscis extension reflex	Lk mutant<br>LK > TNT<br>Lkr mutant	Decreased<br>Decreased<br>Increased	This study<br>This study<br>This study
Blue dye feeding	Lk and Lkr mutants	No effect	This study
CAFE assay	Lk and Lkr mutants<br>Lk > TrpA1<br>Lk; $Gal80^{ts}$ > Ork<br>Lk > Lk RNAi<br>Lk and Lkr mutants	Decreased<br>Decreased<br>Decreased<br>No effect<br>Up to 48h: increased<br>After 48h: decreased	This study<br>[2]<br>[2]<br>[1]<br>[3]
Negative geotaxis	Lk and Lkr mutants	No effect	This study
Locomotor activity	Lk and Lkr mutants<br>Lk > Shi^{ts}<br>Lk > Kir2.1	Decreased<br>No effect<br>Decreased	This study<br>[4]<br>[4]
Metabolic rate	Lk and Lkr mutants	Decreased	This study
DILP2 peptide levels	Lk mutant<br>Lkr mutant	No effect<br>Increased	This study<br>This study
DILP3 peptide levels	Lk and Lkr mutants	Increased	This study
DILP5 peptide levels	Lk and Lkr mutants	No effect	This study
DILP2 transcript	DILP2 > Lkr RNAi	No effect	This study
DILP3 transcript	DILP2 > Lkr RNAi	Increased [b]	This study
DILP5 transcript	DILP2 > Lkr RNAi	Decreased [c]	This study

[a] Increased starvation survival observed for flies maintained as adults on normal, and high sugar and high protein diet only.
[b] *DILP3* transcript is upregulated in flies maintained as adults on normal, and high sugar and high protein diet only.
[c] *DILP5* transcript is down regulated in flies maintained as adults on normal diet.

1. Zandawala M, Marley R, Davies SA, Nässel DR. Characterization of a set of abdominal neuroendocrine cells that regulate stress physiology using colocalized diuretic peptides in *Drosophila*. Cell Mol Life Sci. 2018;75:1099–115. doi: 10.1007/s00018-017-2682-y. PubMed PMID: 29043393.
2. Liu Y, Luo J, Carlsson MA, Nässel DR. Serotonin and insulin-like peptides modulate leucokinin-producing neurons that affect feeding and water homeostasis in *Drosophila*. J Comp Neurol. 2015;523(12):1840-63. Epub 2015/03/04. doi: 10.1002/cne.23768. PubMed PMID: 25732325.
3. Al-Anzi B, Armand E, Nagamei P, Olszewski M, Sapin V, Waters C, et al. The leucokinin pathway and its neurons regulate meal size in *Drosophila*. Curr Biol. 2010;20(11):969-78. Epub 2010/05/25. doi: S0960-9822(10)00517-8 [pii]10.1016/j.cub.2010.04.039. PubMed PMID: 20493701; PubMed Central PMCID: PMC2896026.
4. Cavey M, Collins B, Bertet C, Blau J. Circadian rhythms in neuronal activity propagate through output circuits. Nat Neurosci. 2016;19(4):587-95. doi: 10.1038/nn.4263. PubMed PMID: WOS:000372909800016.
