# Supplementary figures and images for: Modulation of Drosophila post-feeding physiology and behavior by the neuropeptide leucokinin
Source: PLoS Genet. 2018 Nov 20;14(11):e1007767. doi: 10.1371/journal.pgen.1007767 (PMC6245514; doi:10.1371/journal.pgen.1007767)

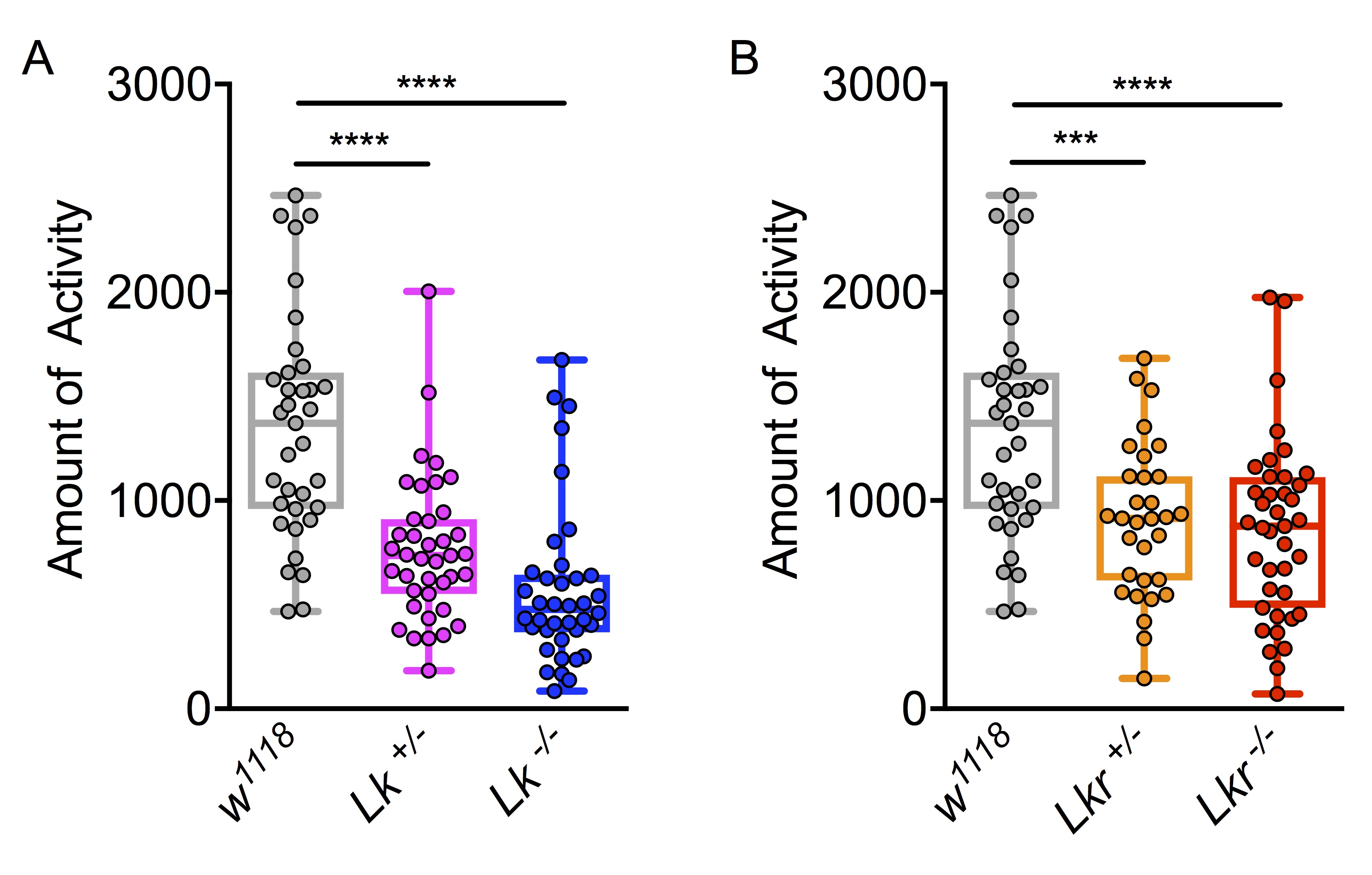

Supplement: S1 Fig — Total locomotor activity of single flies measured over 24 hours is lowered for homozygous and heterozygous (A) Lk and (B) Lkr mutants. The activity was monitored using a standard Drosophila Activity Monitor (DAMS). (*** p < 0.001, **** p < 0.0001, as assessed by one-way ANOVA). (JPG) [file pgen.1007767.s004.jpg]

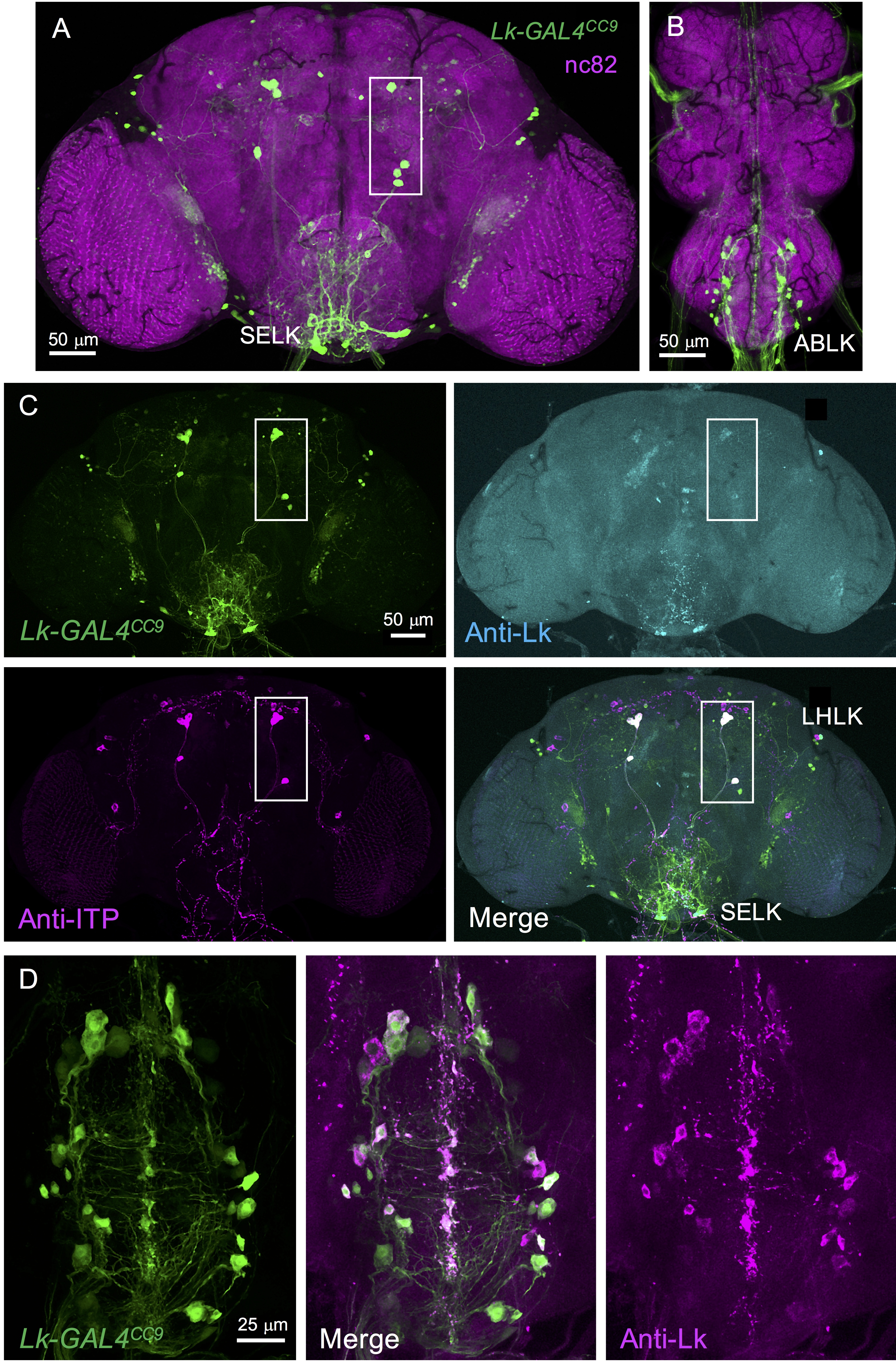

Supplement: S2 Fig — Lk-GAL4CC9 drives GFP (pJFRC81-10xUAS-Syn21-myr::GFP-p10) expression in the adult (A) brain and (B) ventral nerve cord (VNC). SELK, subesophageal LK neurons; ABLK, abdominal LK neurons. Lk-GAL4CC9 also drives GFP expression in four pairs of neurons in the brain (indicated by the white box). (C) These four pairs of neurons display very weak LK-immunoreactivity and are positive for ion transport peptide-immunoreactivity. GFP expression also colocalizes with anti-LK staining in the SELKs and lateral horn LK neurons (LHLK). (D) Lk-GAL4CC9 drives GFP expression in ABLKs (labeled with anti-LK antiserum) in the VNC. (JPG) [file pgen.1007767.s005.jpg]

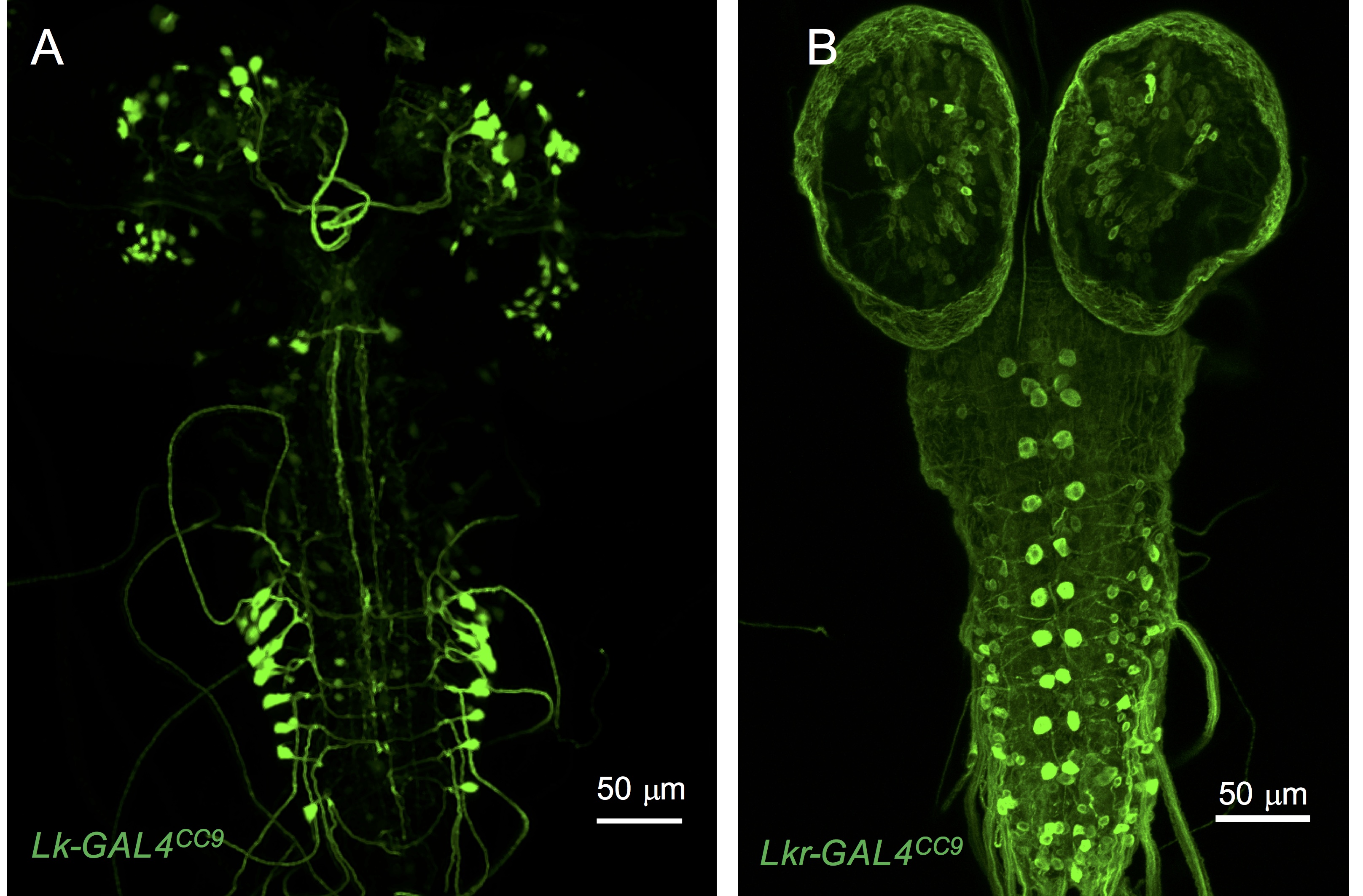

Supplement: S3 Fig — (A) Lk-GAL4CC9 drives GFP (pJFRC81-10xUAS-Syn21-myr::GFP-p10) expression in neurosecretory cells in the larval brain and ventral nerve cord (NVC). (B) Lkr-GAL4CC9 drives GFP (UAS-mCD8;;GFP) expression in larval CNS. Note the GFP expression in motor neurons in the VNC. (JPG) [file pgen.1007767.s006.jpg]

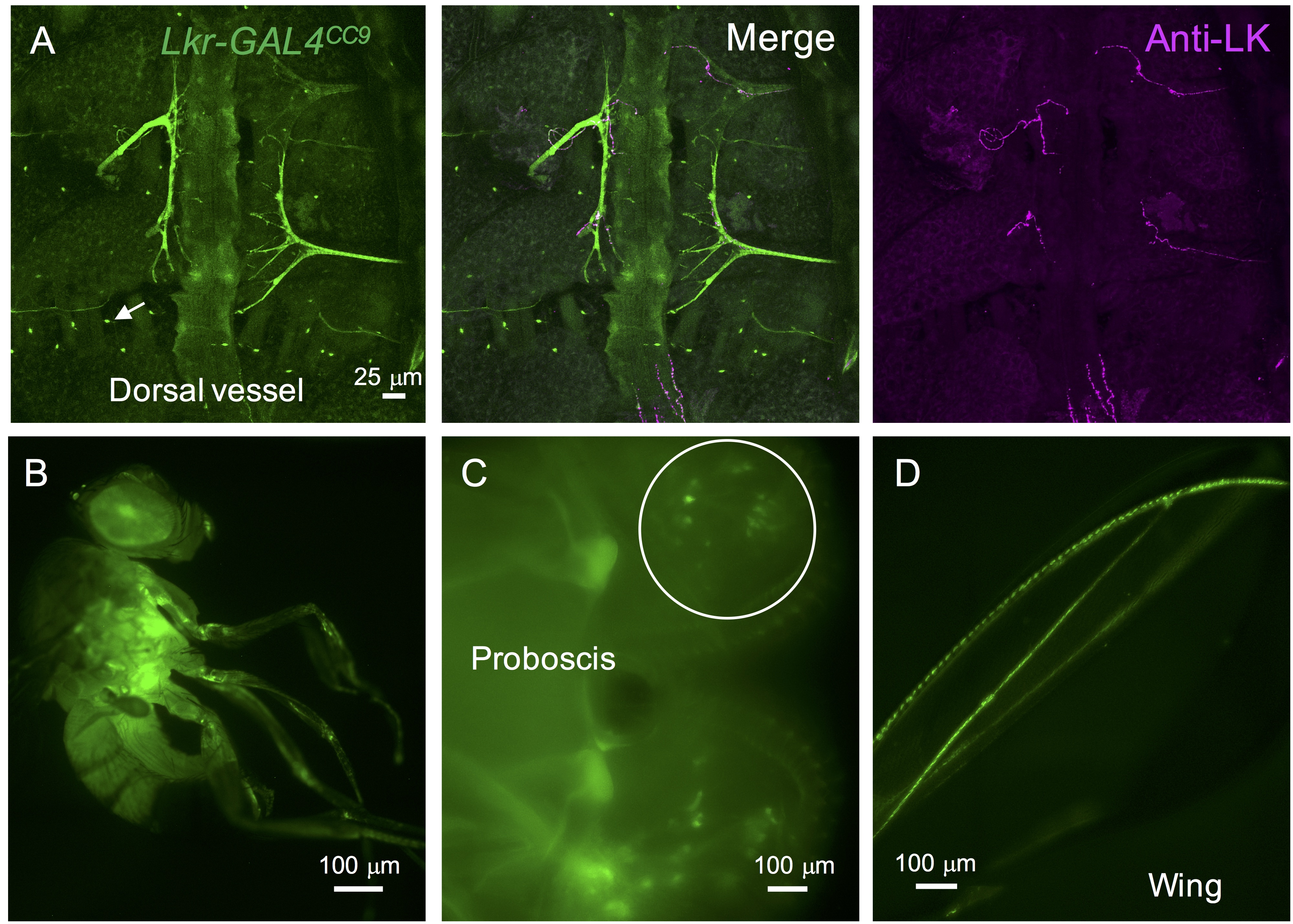

Supplement: S4 Fig — Lkr-GAL4CC9 drives GFP (pJFRC81-10xUAS-Syn21-myr::GFP-p10) expression in the adult (A) dorsal vessel and peripheral neurons (indicated by an arrow), (B) legs, (C) proboscis, and (D) wings. Note the expression of Lkr in nerve fibers closely associated with the anti-LK immunostaining in (A). (JPG) [file pgen.1007767.s007.jpg]

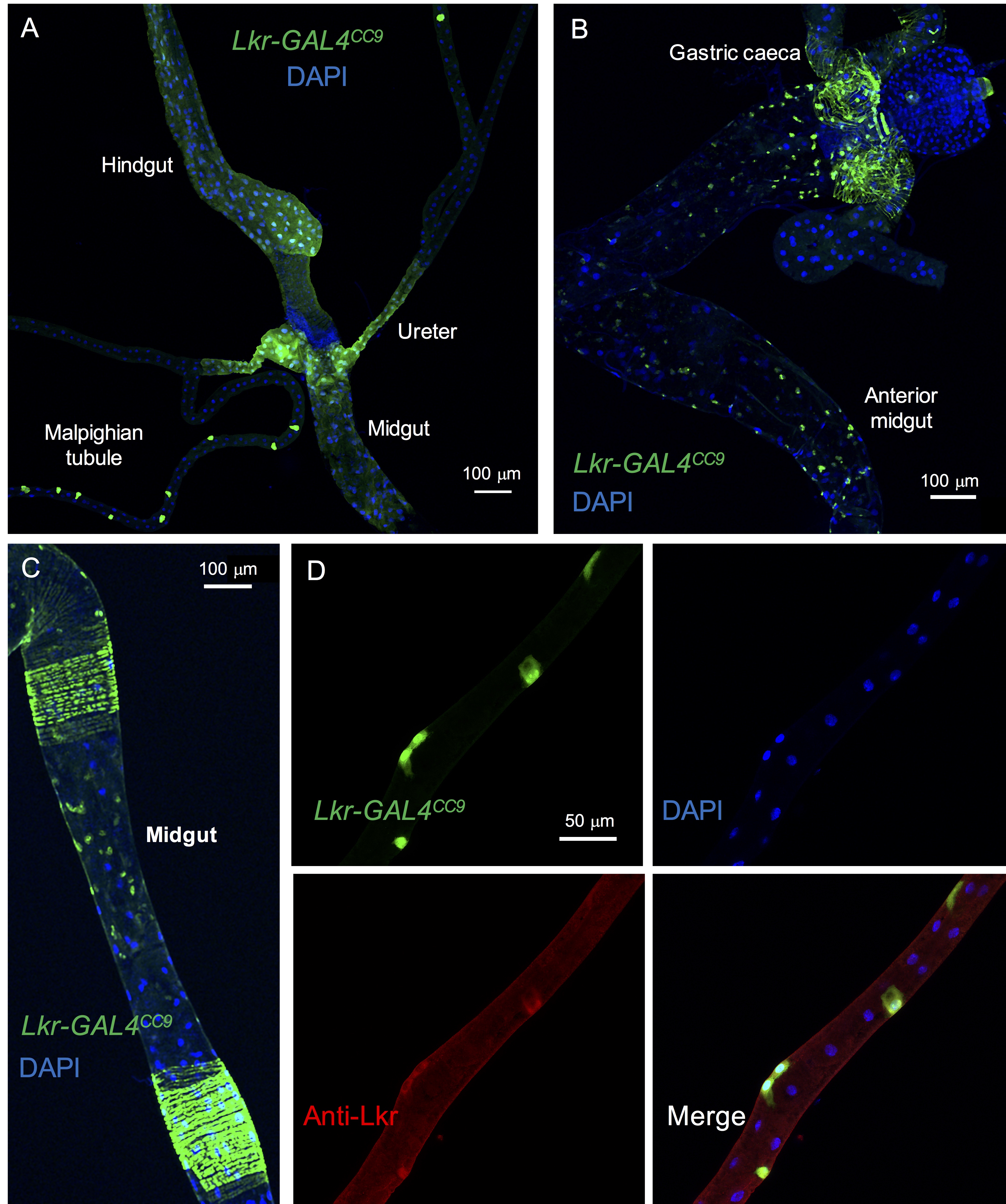

Supplement: S5 Fig — Lkr-GAL4CC9 drives GFP (pJFRC81-10xUAS-Syn21-myr::GFP-p10) expression in the larval (A) gut, (B) gastric caeca and anterior midgut, (C) midgut, and (D) anti-DromeLkr-expressing stellate cells in Malpighian tubules. Nuclei in all the preparations have been stained with DAPI (blue). (JPG) [file pgen.1007767.s008.jpg]

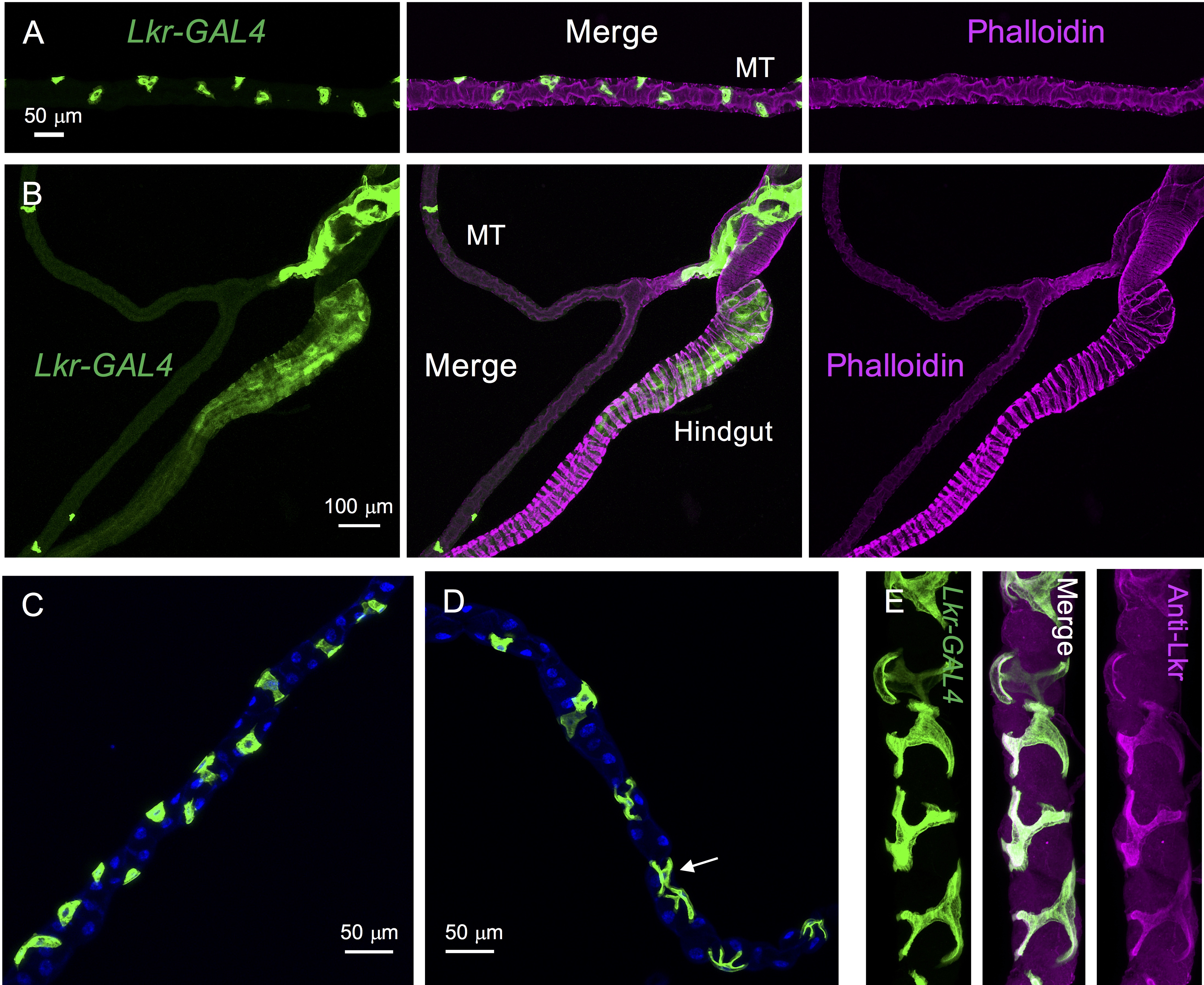

Supplement: S6 Fig — Lkr-GAL4 drives GFP (pJFRC29-10xUAS-myr::GFP-p10) expression in (A) the larval stellate cells of Malpighian tubules, (B) larval hindgut, and (C-E) adult stellate cells (labeled with anti-DromeLkr antiserum). Note that the adult stellate cells can be (C) cuboidal or (D) star-shaped (indicated by an arrow). (JPG) [file pgen.1007767.s009.jpg]

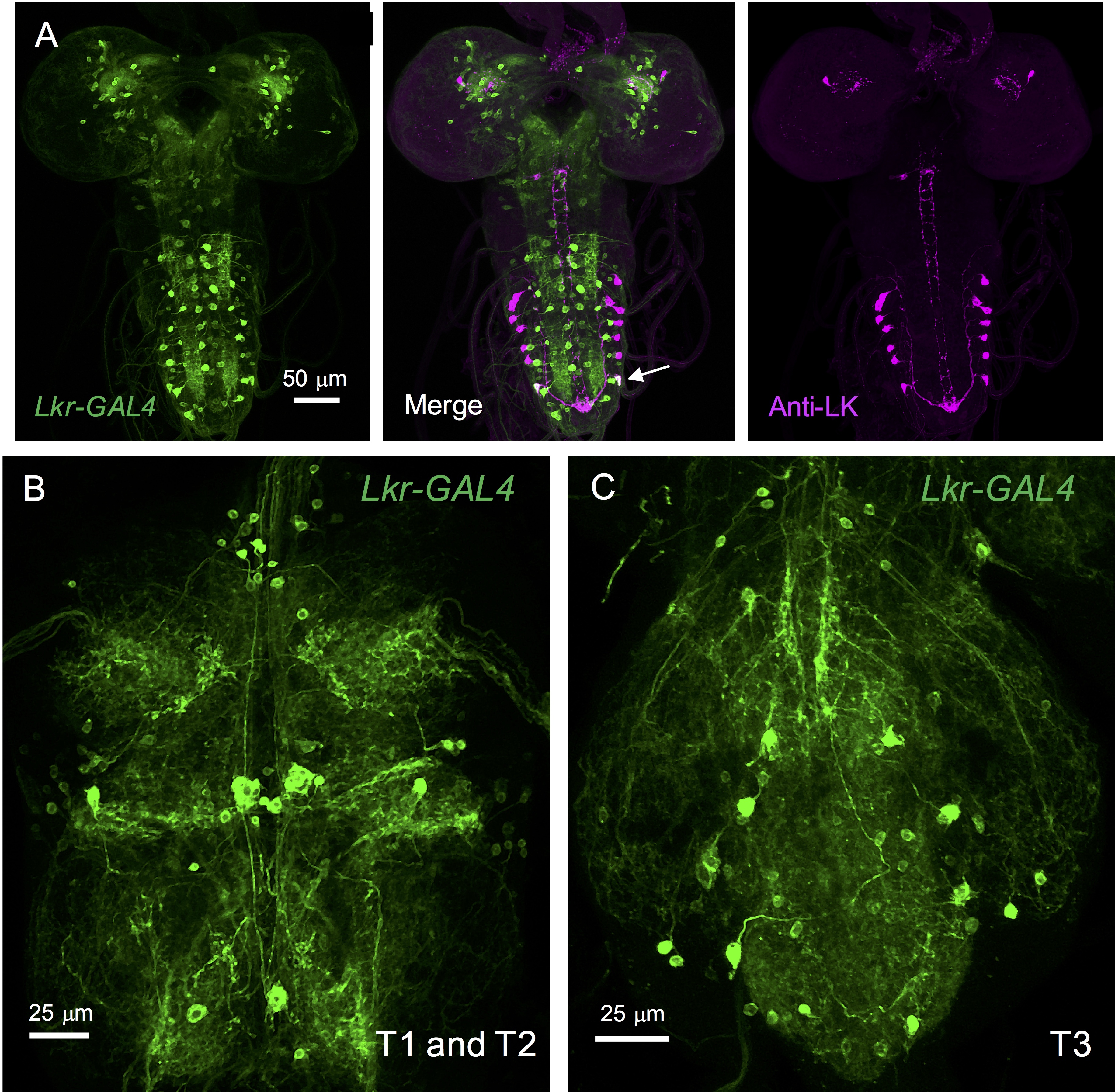

Supplement: S7 Fig — (A) Lkr-GAL4 drives GFP expression in several neurons of the larval CNS, including a pair of abdominal Lk neurons stained with anti-Lk antiserum (indicated by arrow). In adults, Lkr-GAL4 drives GFP expression in (B) T1 and T2 thoracic neuromeres and (C) T3 thoracic neuromere. (JPG) [file pgen.1007767.s010.jpg]

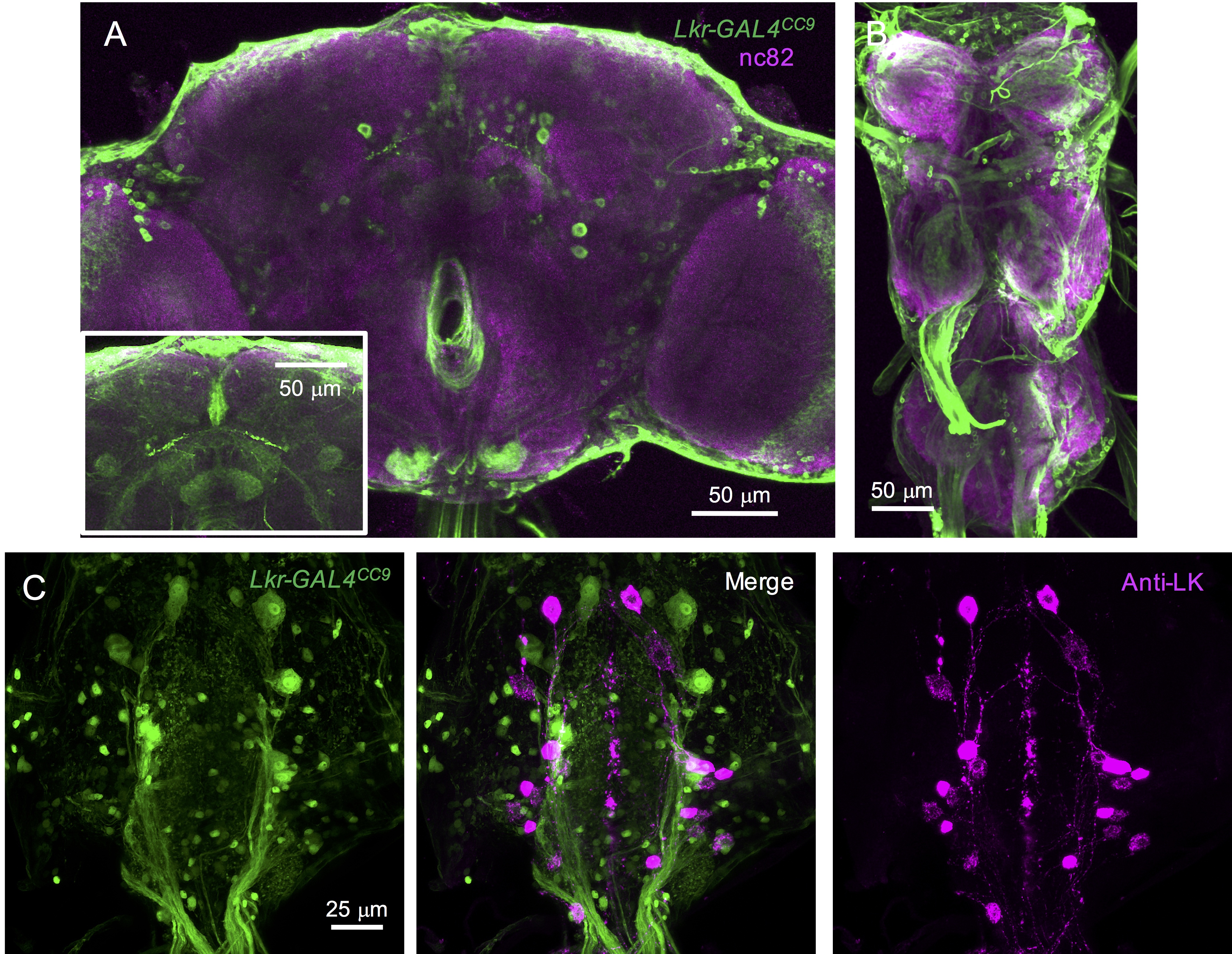

Supplement: S8 Fig — Lkr-GAL4CC9 drives GFP (UAS-mCD8;;GFP) expression in (A) the brain and (B) ventral nerve cord. The inset in (A) represents a smaller Z-stack, which shows GFP expression in the fan-shaped body. These preparations were counterstained with anti-nc82 antiserum. (C) Lkr-GAL4CC9 drives GFP (pJFRC81-10xUAS-Syn21-myr::GFP-p10) expression in neurons of the abdominal ganglia that do not express LK. (JPG) [file pgen.1007767.s011.jpg]

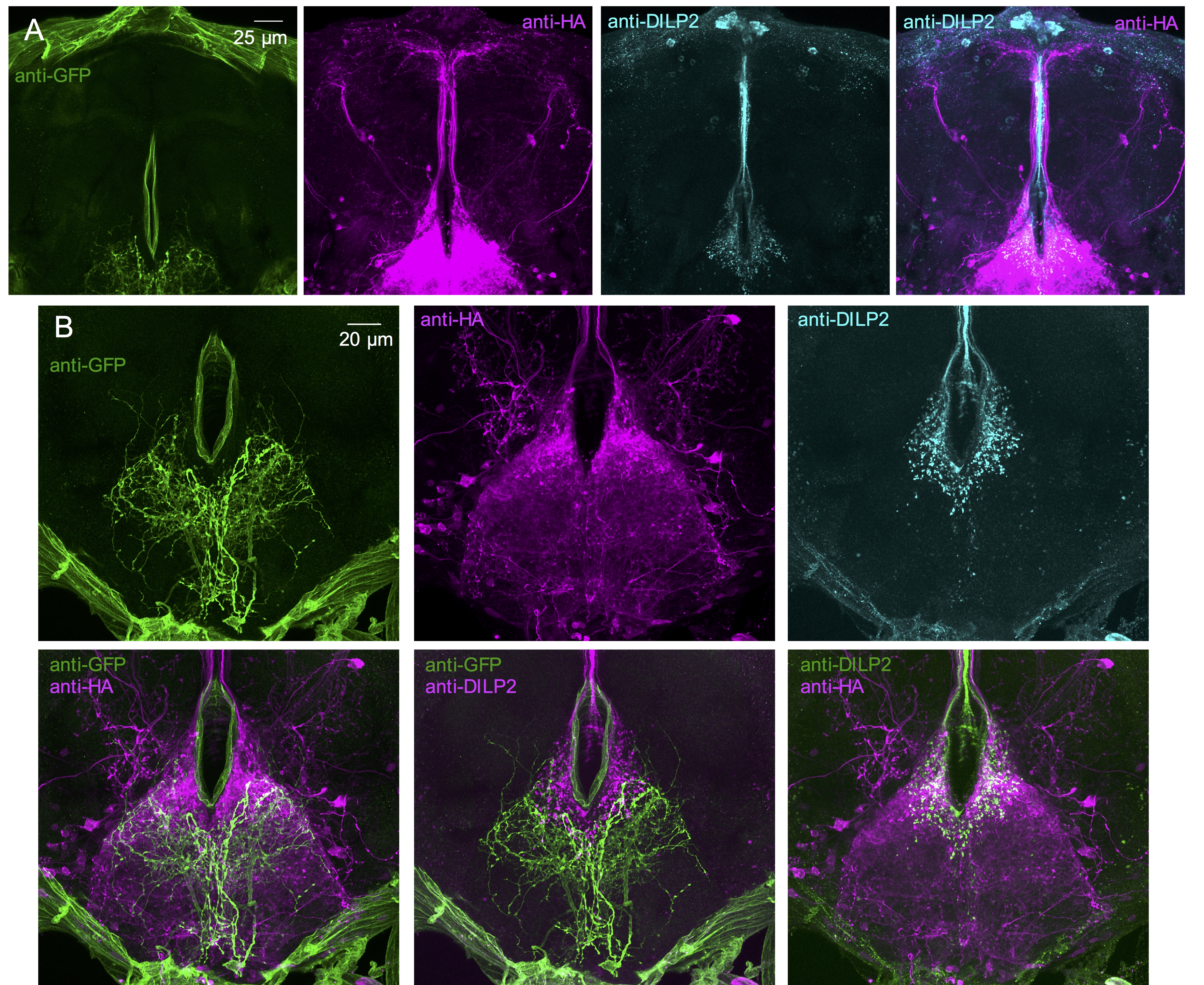

Supplement: S9 Fig — (A) Expression of trans-Tango components [31] using Lk-GAL4 (from P. Herrero) generates a presynaptic signal (labeled with anti-GFP antibody) in the subesophageal ganglion (SEG) and a postsynaptic signal (labeled with anti-HA antibody) in the SEG and pars intercerebralis, which does not colocalize with insulin-producing cells or their axons (labeled with anti-DILP2 antibody). (B) Higher magnification of the SEG showing the presynaptic and postsynaptic signals and the lack of colocalization with anti-DILP2 staining. (JPG) [file pgen.1007767.s012.jpg]

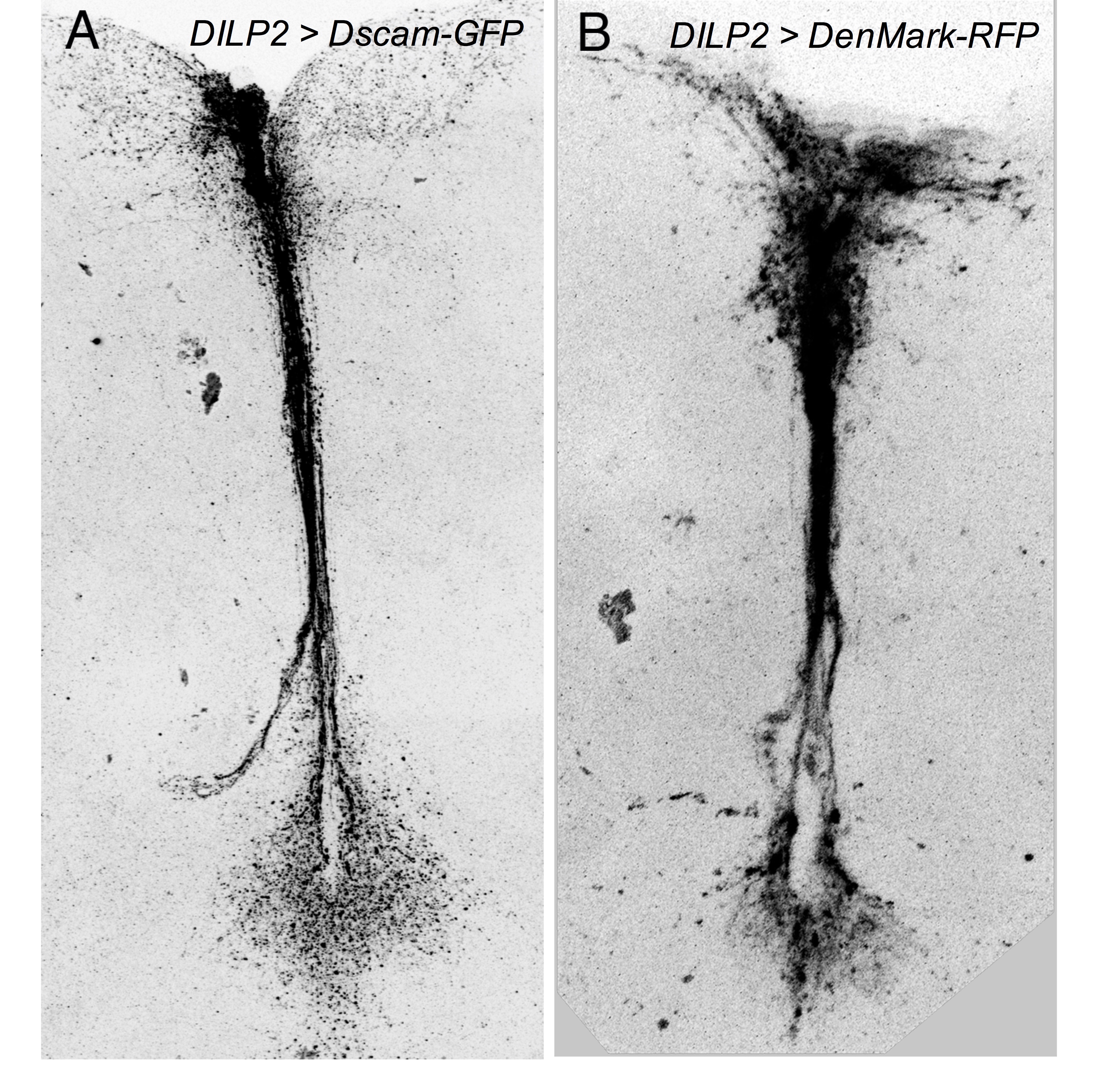

Supplement: S10 Fig — Using dendrite-directed UAS constructs, fluorescent labeling can be seen in IPC processes in pars intercerebralis and tritocerebrum/subesophageal zone, shown in inverted images. (A) DILP2-GAL4 driven Dscam-GFP and (B) DILP2-GAL4 driven DenMark-RFP. These images were kindly provided by Dr. Yiting Liu. (JPG) [file pgen.1007767.s013.jpg]

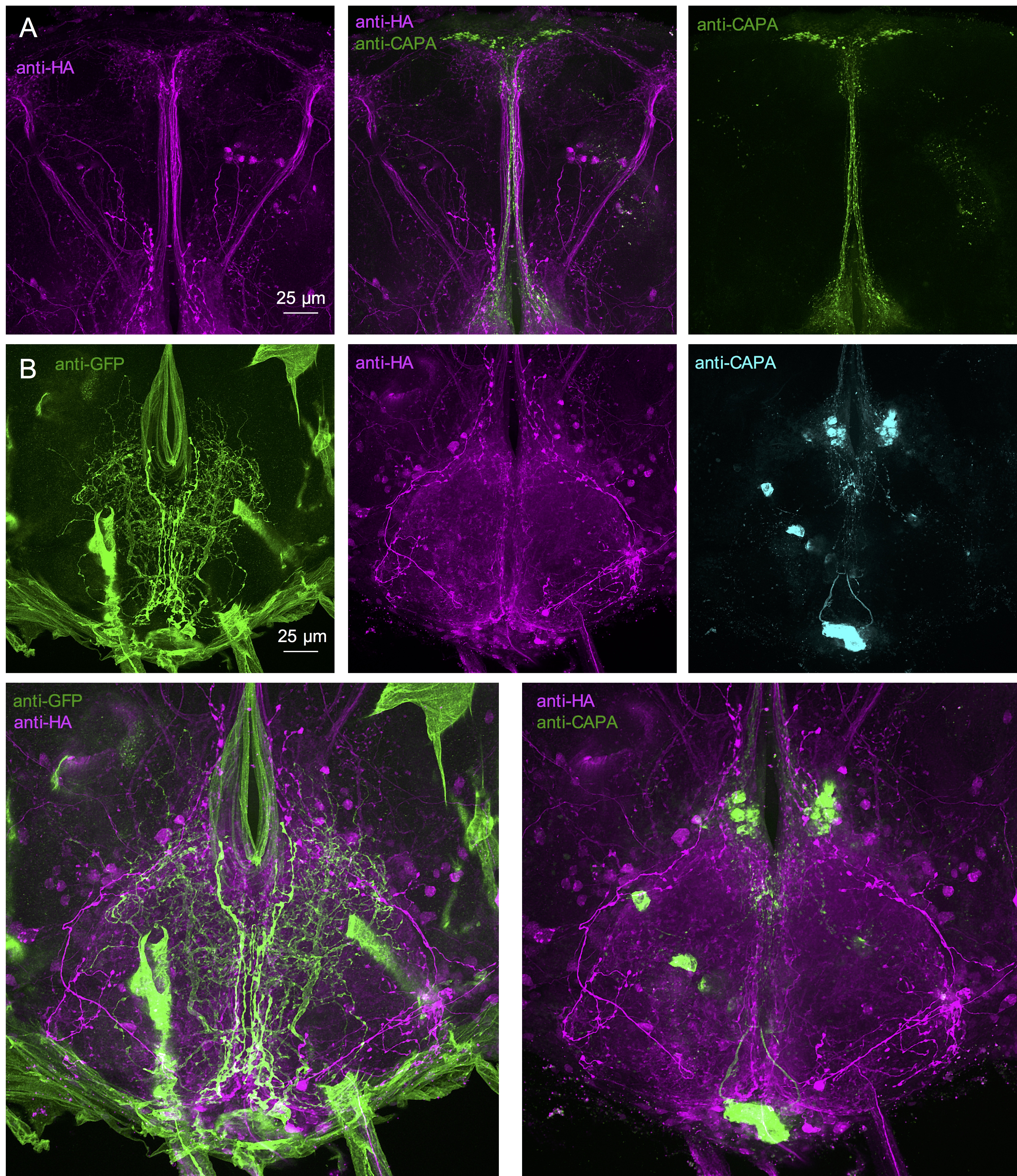

Supplement: S11 Fig — (A) Expression of trans-Tango components [31] using Lk-GAL4 generates a post-synaptic signal (labeled with anti-HA antibody) in the tritocerebrum and pars intercerebralis which does not colocalize with CAPA/hugin axons (labeled with anti-CAPA antibody). (B) Higher magnification of the subesophageal ganglion showing the pre-synaptic and post-synaptic signals and the lack of colocalization with anti-CAPA staining. (JPG) [file pgen.1007767.s014.jpg]

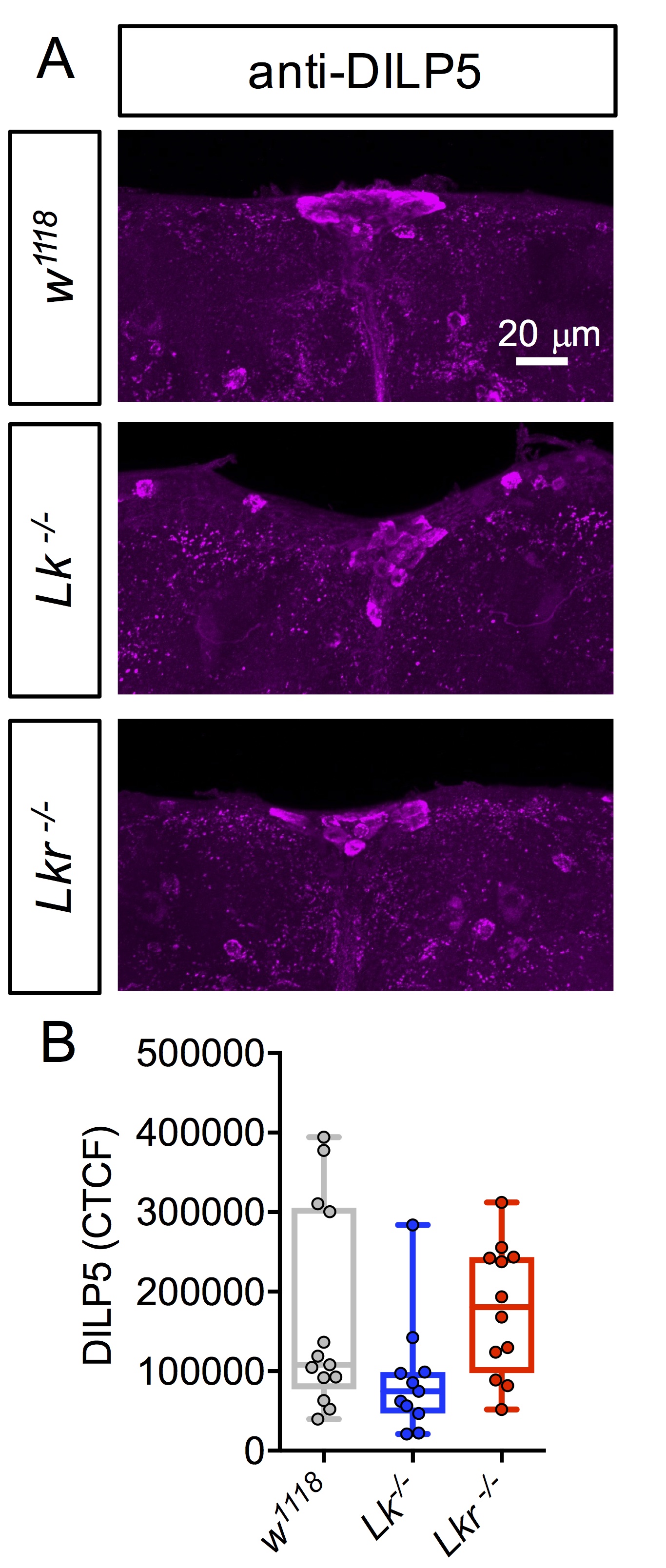

Supplement: S12 Fig — (A) Lk and Lkr homozygous mutants do not display any difference in DILP5 immunoreactivity in insulin-producing cells (IPCs) of the adult brain. (B) Fluorescence intensity measurement of IPCs shows no difference in DILP5 immunoreactivity in Lk and Lkr mutant flies compared to control flies. CTCF, corrected total cell fluorescence. (JPG) [file pgen.1007767.s015.jpg]

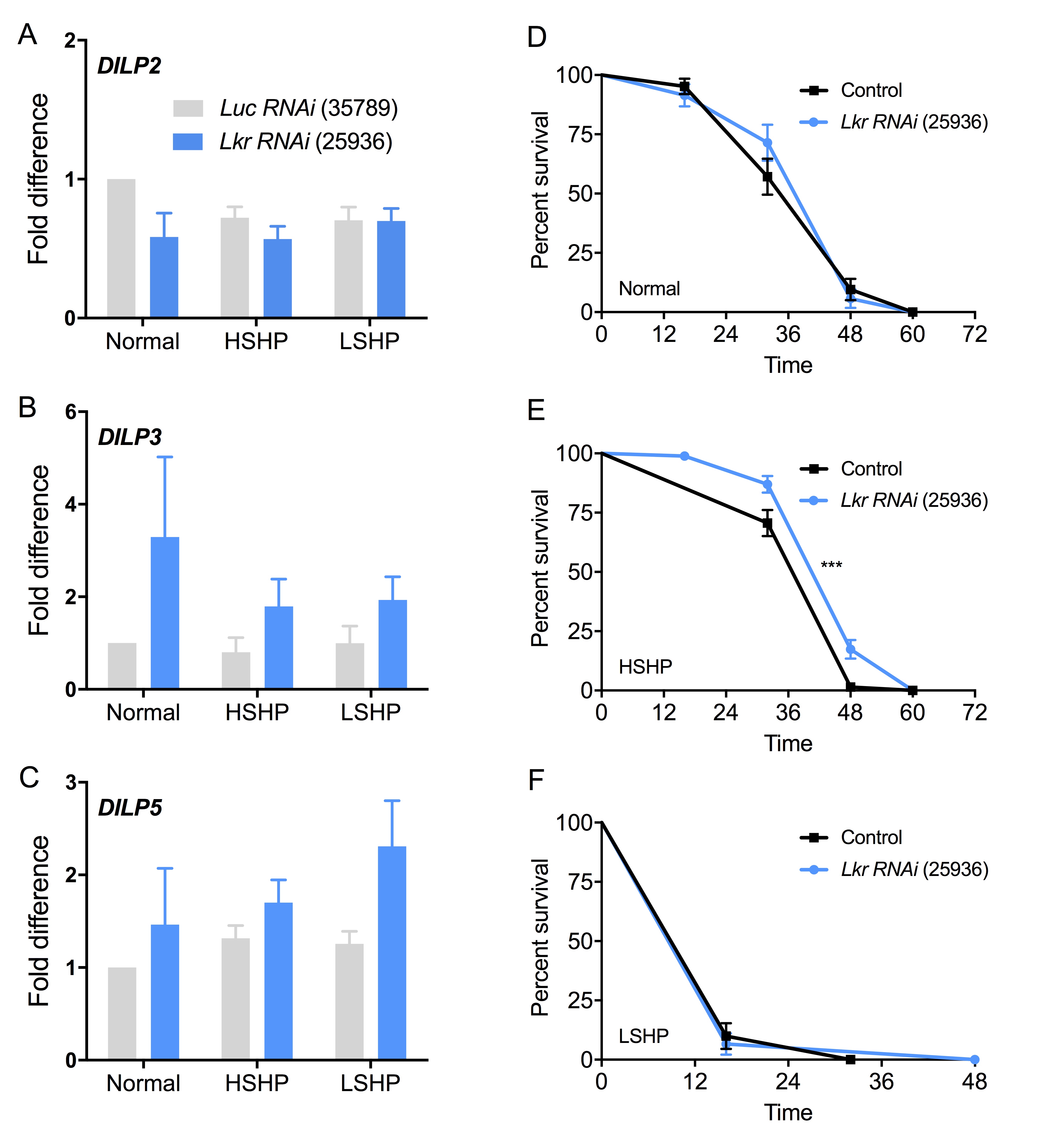

Supplement: S13 Fig — Quantitative PCR shows no difference in (A) DILP2, (B) DILP3, and (C) DILP5 transcript levels between control flies (DILP2>Luciferase) and flies with Lkr knockdown in insulin-producing cells (IPCs) (DILP2>Lkr-RNAi-#1 (BL#25936) that were reared as adults on normal diet, high sugar and high protein (HSHP) diet or low sugar and high protein (LSHP) diet. Flies maintained as adults on (E) HSHP diet show increased starvation resistance whereas flies maintained on (D) normal diet and (F) LSHP diet have similar survival under starvation compared to control flies. For graphs D-F, data are presented in survival curves and the error bars represent standard error (*** p < 0.001, as assessed by Log-rank (Mantel-Cox) test). (JPG) [file pgen.1007767.s016.jpg]
